# Concurrent Inhibition of AKT and GLUT1 Reveals Endpoint-Specific Effects on Glucose Metabolism and Cell Death in HepG2 Hepatocyte-Derived Cells

**DOI:** 10.3390/biomedicines14092133

**Published:** 2026-09-21

**Authors:** Abutaleb Asiri, Abeer Al Otaibi, Munazzah Tasleem, Sindiyan Al Shaikh Mubarak, Muwadah Al Said, Yara Al Mihmadi, Badr Al Subei, Abdulaziz Asiri, Ali Al Qarni, Ahmed Bakillah

**Affiliations:** 1 King Abdullah International Medical Research Center (KAIMRC), Eastern Region, Al Ahsa 36428, Saudi Arabia; asiriabu@kaimrc.med.sa (A.A.); otaibiabe@kaimrc.med.sa (A.A.O.); alshaikhmubaraks@kaimrc.med.sa (S.A.S.M.); saidm@kaimrc.med.sa (M.A.S.); mihmadiy@kaimrc.med.sa (Y.A.M.); alsubeib@kaimrc.med.sa (B.A.S.); 2 Division of Medical Research Core, King Saud bin Abdulaziz University for Health Sciences (KSAU-HS), Al Ahsa 36428, Saudi Arabia; 3 King Abdulaziz Hospital, Ministry of National Guard-Health Affairs (MNG-HA), Al Ahsa 36428, Saudi Arabia; qarniaa@mngha.med.sa; 4 Department of Public Health, College of Applied Medical Sciences in Al-Namas, University of Bisha, Al-Namas City 67714, Saudi Arabia; mtaslem@ub.edu.sa; 5 Department of Medical Laboratory Sciences, College of Applied Medical Sciences, University of Bisha, 255, Al Nakhil, Bisha 67714, Saudi Arabia; amfasiri@ub.edu.sa

**Keywords:** hepatocyte, HepG2, insulin signaling, AKT, GLUT1, cell proliferation, apoptosis, glucose metabolism, glycogen, metabolism, PI3K/AKT pathway

## Abstract

**Background**: Glucose handling in hepatocyte-derived cells integrates insulin-responsive AKT signaling with GLUT1-mediated glucose transport, jointly regulating substrate trafficking, glycogen synthesis, and cell survival. MK-2206, a potent allosteric AKT inhibitor, and BAY-876, a highly selective GLUT1 inhibitor, represent targeted approaches to disrupt these processes. However, the consequences of simultaneous inhibition of both nodes in hepatocyte-derived cells remain incompletely understood. **Objective**: This study aims to evaluate the impact of simultaneous AKT inhibition and GLUT1 blockade on target engagement, cell viability, proliferation, cell death, glucose uptake, and glycogen storage in HepG2 cells. **Methods**: HepG2 cells were treated with MK-2206, BAY-876, or their combination under basal hyperglycemic and acute insulin-challenged conditions. Multi-model synergy, AKT/GLUT1 pharmacological effects, cell viability, proliferation, cell death, glucose uptake, and glycogen storage were assessed, alongside exploratory molecular docking analyses of both inhibitors against their respective targets. **Results**: Dual AKT and GLUT1 inhibition produced combination-specific reductions in cell viability and showed a pattern of concurrent antiproliferative effects and increased Annexin V/PI positivity without detectable caspase-3 activation under insulin-challenged conditions. Furthermore, the dual inhibition of AKT signaling and GLUT1-mediated glucose uptake produced combination-specific reductions in basal glycogen content, while glucose uptake reductions showed AKT-dominant patterns without significant combination superiority over MK-2206 alone under either metabolic state. Exploratory molecular docking of MK-2206 and BAY-876 against AKT1 and SLC2A1/GLUT1, respectively, generated computationally predicted binding poses consistent with the established binding modes of these inhibitors. **Conclusions**: Dual AKT/GLUT1 targeting produces endpoint-specific patterns of pharmacological interaction on insulin-responsive metabolism, proliferation, and cell death in HepG2 hepatocyte-derived cells, distinguishing combination-specific effects from AKT-driven effects. These findings support an endpoint-specific pharmacological framework in which AKT signaling and BAY-876-mediated GLUT1 pharmacological effects jointly influence glucose handling and cell fate in a hepatocyte-derived model.

## 1. Introduction

Hepatic glucose handling is a tightly regulated process that integrates insulin-responsive signaling with substrate transport to maintain systemic glucose homeostasis [1,2]. Disruption of this process contributes to a range of pathological hepatic states, including insulin resistance (IR), metabolic dysfunction-associated steatotic liver disease (MASLD), type 2 diabetes mellitus (T2DM), and hepatocellular carcinoma (HCC) [3,4,5,6,7,8]. IR is a result of impaired response of target tissues to insulin stimulation, leading to compensatory high insulin levels in the blood, referred to as hyperinsulinemia, a hallmark feature of obesity and T2DM that fosters the progression toward HCC [1,9,10,11]. In hepatocytes, sustained hyperinsulinemia promotes lipid accumulation and drives the transition from steatosis to HCC [6,10,12]. The resulting metabolic environment, characterized by hyperglycemia and elevated insulin signaling, substantially alters hepatocyte glucose handling [10,13]. This metabolic dysregulation provides a clinically relevant context for studies of hepatocyte glucose handling under hyperglycemia and elevated insulin signaling.

A central player in insulin-mediated hepatic metabolism is the phosphatidylinositol 3-kinase (PI3K)/protein kinase B (AKT) signaling pathway, which regulates metabolic homeostasis and insulin sensitivity [1,2]. Insulin stimulates glycogen storage by activating the PI3K/AKT pathway, which ultimately facilitates glucose transporter-mediated uptake and inactivates glycogen synthase kinase 3 (GSK3) [14]. Activation of AKT signaling promotes glucose trafficking and utilization through downstream effectors, including glucose transporters (GLUTs) [1,15]. Dysregulation of AKT signaling is associated with altered hepatic states, including inflammation, fibrosis, disease progression, and poor prognosis in chronic liver disease [16,17]. Allosteric AKT inhibitors such as MK-2206 have emerged as valuable tools for dissecting insulin-responsive AKT signaling and modulating downstream metabolic and proliferative pathways in hepatocyte-derived models [17,18,19].

Glucose transporter-1 (GLUT1) is a major glucose transporter that facilitates basal glucose uptake across the plasma membrane and plays a crucial role in cellular energy metabolism, particularly in cells with increased glycolytic demand [20,21]. While GLUT2 is the predominant facilitative glucose transporter in normal hepatocytes, the HepG2 cell line exhibits a distinctly altered transporter expression profile characteristic of its transformed phenotype, with upregulated GLUT1 and reduced GLUT2 expression relative to primary hepatocytes [22]. Quantitative analysis of GLUT family members has established GLUT1 as the most highly expressed glucose transporter, with functional studies confirming that GLUT1 is the major mediator of glucose influx in HepG2 cells [22]. Consistent with this, GLUT1 overexpression is frequently observed in various pathological states associated with altered glucose handling, underscoring its importance in cellular energy metabolism [23,24]. In HepG2 cells, GLUT1 expression has also been closely linked to proliferative capacity [25]. The therapeutic potential of targeting AKT signaling and GLUT1-mediated glucose transport has been explored across multiple cancer contexts. In breast cancer, MK-2206 combined with the GLUT1 inhibitor WZB117 exerts synergistic cytotoxic effects by inhibiting AKT phosphorylation and inducing DNA damage, with subsequent work extending these observations with additional GLUT1 inhibitors [26,27]. In lung cancer, both AKT inhibition combined with glucose metabolism disruption and GLUT1 inhibitor combined with EGFR inhibitors have demonstrated synergistic anti-tumor effects [28,29]. Additionally, WZB117 has been shown to inhibit cell growth as a single agent in non-small cell lung cancer models [30]. These findings collectively establish AKT and GLUT1 targeting as a broader anti-tumor pharmacological strategy across multiple cancer types. However, the consequences of dual AKT/GLUT1 pharmacological targeting in hepatocyte-derived cells, particularly under insulin-responsive conditions, remain incompletely characterized. Among reported GLUT1 inhibitors, BAY-876 is a highly selective inhibitor that effectively blocks GLUT1-mediated glucose uptake, with minimal activity against GLUT2 and GLUT3, enabling more precise interrogation of GLUT1-specific contributions to hepatocyte glucose handling [31,32,33,34].

Despite the established roles of AKT signaling and GLUT1-mediated glucose transport in hepatic metabolism, the consequences of simultaneously inhibiting both nodes on insulin-responsive glucose handling, glycogen storage, and cell fate in hepatocyte-derived cells under basal hyperglycemic and acute insulin-challenged conditions remain incompletely understood [16,35]. While AKT and GLUT1 combination targeting has been previously investigated in other cancer contexts, the pharmacological consequences of combining MK-2206 with the highly selective GLUT1 inhibitor BAY-876 in HepG2 hepatocyte-derived cells under basal and acute insulin-challenged conditions have not been characterized. HepG2 cells provide an advantageous model for such investigations, as they retain the robust insulin-responsive PI3K/AKT signaling and glycogen synthesis machinery characteristic of human hepatocytes, features that are commonly lost in most dedifferentiated hepatic cell lines [36,37,38]. Thus, this study aims to determine whether dual inhibition of AKT using the allosteric AKT inhibitor MK-2206 and GLUT1 using the selective inhibitor BAY-876 produces synergistic effects on insulin-responsive metabolism in HepG2 hepatocyte-derived cells under basal hyperglycemic and acute insulin-challenged conditions, and to delineate the resulting alterations in AKT/GLUT1 target engagement, glucose uptake, glycogen storage, proliferation, and cell death. To complement the cellular findings, molecular docking analyses of MK-2206 against AKT1 and BAY-876 against SLC2A1/GLUT1 were performed as an exploratory in silico approach to provide structural context for plausible interactions of these inhibitors. By integrating pharmacological, metabolic, and cell death analyses within a single hepatocyte model, alongside exploratory structural docking, the present study provides an endpoint-specific pharmacological framework for characterizing the interaction between insulin-responsive AKT signaling and GLUT1-targeted pharmacological effects in hepatocyte-derived cell glucose handling. We hypothesize that concurrent inhibition of AKT signaling and GLUT1 will disrupt insulin-responsive glucose handling and induce cell death by simultaneously suppressing AKT activation and limiting GLUT1-mediated glucose transport, with the exploratory docking analyses providing complementary structural context for the established inhibitor–target interactions.

## 2. Materials and Methods

### 2.1. Cells and Reagents

HepG2 cell line used in this study was obtained from ATCC (ATCC ID: HB-8065). Dimethyl Sulfoxide (DMSO) was obtained from Thermo Fisher Scientific (Eugene, OR, USA, Cat. No. D12345). The MTT (3-(4,5-dimethyl-2-thiazolyl)-2,5-diphenyl-tetrazolium bromide) was purchased from HiMedia (Maharashtra, India, Cat. No. CCK003). Compounds were obtained from MedChemExpress, Monmouth Junction, NJ, USA: MK-2206 (Cat No. HY-10358) and BAY-876 (Cat. No. HY-100017). All antibodies used in this study were purchased from Cell Signaling Technology (Danvers, MA, USA): GAPDH (Cat. No. 2118), Total AKT (Cat. No. 9272), p-AKT (Cat. No. 4058S), mTOR (Cat. No. 2983), p-mTOR (Cat. No. 5536), GLUT1 (Cat. No. 12939), p70S6K (Cat. No. 9202), p-p70S6K (Cat. No. 9204), IRS-1 (Cat. No. 2390), p-IRS-1 (Cat. No. 2381), AS160 (Cat. No. 2670), p-AS160 (Cat. No. 4288), ERK1/2 (Cat. No. 9102), p-ERK1/2 (Cat. No. 4370), BCL-2 (Cat. No. 4223), BAK (Cat. No. 12150), BID (Cat. No. 2002), Bax (Cat. No. 5023), except Ki-67 (Cat. No. AB-M-105) and PCNA (AB-M-110) from MOLEQULE-ON (New Lynn, Auckland, New Zealand), and GLUT2 (Cat. No. SL0351R) from SUNLONG (Binging District, Hangzhou, Zhejiang, China).

### 2.2. Cell Culture

HepG2 cell line was cultured in Dulbecco’s Modified Eagle Medium (DMEM; Thermo Fisher Scientific, Waltham, MA, USA) containing 25 mM glucose (High-glucose), supplemented with 10% fetal bovine serum (FBS), 1% Penicillin-Streptomycin, and 1% L-Glutamine (200 mM). This media formulation represents the standard high-glucose DMEM composition (4.5 g/L) routinely used for HepG2 cell culture and was selected to provide standardized substrate availability across experimental groups rather than to model physiological hyperglycemic conditions. Cells were maintained in a humidified incubator with 5% CO_2_ at 37 °C [39].

### 2.3. Determination of Half-Maximal Inhibitory Concentration (IC_50_) by MTT Assay

To determine the IC_50_ of MK-2206 and BAY-876 in HepG2 cells, cells were harvested during exponential growth, counted using an automated cell counter (Countess 3, Invitrogen, Bothell, WA, USA), and seeded at 2000 cells per well in a 96-well plate in accordance with our published study [39]. After allowing the cells to adhere and grow for 24 h, the culture medium in each well was replaced with an equal volume (100 µL) of culture medium containing MK-2206 or BAY-876 at the indicated concentrations, and the cells were allowed to grow for 48 h before subsequent MTT assay analyses. The 48 h treatment duration was selected based on the established pharmacological considerations for both inhibitors. MK-2206 is an allosteric AKT inhibitor that requires sustained drug exposure to achieve maximal cellular AKT suppression through prevention of membrane recruitment and conformational activation, while BAY-876 selectively inhibits GLUT1-mediated glucose flux, requiring prolonged exposure for downstream metabolic adaptation. This 48 h window is consistent with established literature for kinase inhibitor concentration-response analysis in hepatocyte-derived cell line models and aligns with the temporal scale of secondary cellular responses, including changes in proliferation, metabolic adaptation, and apoptotic engagement [40,41,42]. Each 96-well plate included cells treated with vehicle control (DMSO). MTT assay was performed in accordance with the manufacturer’s protocol. Briefly, 10 µL of MTT was added to each well, including blank controls, and the plates were incubated for 4 h at 37 °C to ensure sufficient formazan formation without saturation, according to established protocols [43,44]. Following incubation, formazan crystals were dissolved in 100 µL of solubilization solution by gentle shaking on a gyratory shaker. Absorbance was measured at 570 nm with a reference wavelength of 630 nm using a microplate reader (Synergy H1, BioTek, Winooski, VT, USA). Cell viability at different concentrations of each inhibitor (MK-2206 and BAY-876) was calculated relative to vehicle control. IC_50_ values were determined by nonlinear regression using a four-parameter logistic (4PL) sigmoidal model in GraphPad Prism v11 (Boston, MA, USA), with 95% confidence intervals derived from the fitted curves. IC_50_ determination was performed with *n* = 3 independent biological experiments.

### 2.4. Synergy Assessment of Drug Combination Effects

Synergy analysis was performed to evaluate the effect of dual inhibition of AKT signaling and GLUT1 on cell viability in HepG2 cells, to select the combination that yields the highest synergy score for downstream signaling, functional, and metabolic analyses. Three concentrations around the IC_50_ were selected for synergy analysis to capture interactions at pharmacologically relevant doses where the drug exerts a meaningful but submaximal effect, thereby enabling detection of additive, synergistic, or antagonistic interactions without complete growth inhibition that could obscure cooperative effects. The analyses of three concentrations (MK-2206 at 6, 8, and 10 µM with BAY-876 at 4, 6, and 8 µM) were tested in a 3 × 3 matrix, including single-agent and vehicle controls in triplicate. The synergy analysis was performed with two independent biological experiments (*n* = 2). After 48 h of treatment, cell viability was measured by MTT, normalized to vehicle controls, and analyzed in SynergyFinder under four models: HSA (Highest Single Agent), Bliss (Bliss independence), Loewe (Loewe additivity), and ZIP (zero interaction potency), to generate synergy scores as described previously [45]. This multi-model approach was applied to provide convergent evidence of pharmacological synergy across different assumptions, with synergy scores > 10 indicating synergy, between −10 and 10 indicating additivity, and <−10 indicating antagonism, based on the widely used SynergyFinder interpretation framework [46,47]. The combination with the highest cross-model synergy was selected for subsequent signaling, functional, and metabolic investigations, and enhanced viability suppression at the selected combination concentrations was further assessed using the ATP-derived CellTiter-Glo viability assay (Section 2.5).

### 2.5. Cell Viability Assessment Under Basal and Acute Insulin-Challenged Conditions

Following the formal SynergyFinder synergy analysis based on MTT measurements (Section 2.4), enhanced viability suppression at the selected combination (10 µM MK-2206 + 6 µM BAY-876) was further assessed using the ATP-derived CellTiter-Glo (CTG) assay with three independent biological experiments (*n* = 3) under basal and acute insulin-challenged conditions. This CTG assessment employed a methodologically distinct functional viability measurement (cellular ATP content vs. mitochondrial reductase activity in the MTT assay), though both endpoints remain dependent on cellular metabolism. HepG2 cells were seeded at 2000 cells per well in a 96-well plate and treated for 48 h with MK-2206 (10 µM), BAY-876 (6 µM), or their combination in high-glucose (25 mM) complete medium. The 48 h endpoint was selected to align with the IC_50_ determination window and capture sustained pharmacological effects on cellular viability. Two metabolic states were evaluated: basal hyperglycemic (without insulin) and an acute insulin-challenged state (with insulin). For the acute insulin-challenged conditions, following 48 h of drug treatment, cells were serum-starved in low-glucose serum-free medium for 4 h, followed by a 15 min acute insulin challenge (250 nM), as described previously [48]. The 250 nM insulin concentration was selected as a supraphysiological pharmacological stimulus to elicit maximal AKT phosphorylation for robust characterization of dual AKT/GLUT1 inhibition effects on insulin-responsive signaling, rather than to model physiological insulin action, insulin resistance, or metabolic liver disease. The 4 h serum starvation period prior to the insulin challenge was applied to ensure a baseline of deactivated insulin signaling activity, allowing the acute insulin response to be measured against a clean signaling baseline activity [49,50]. The 15 min period for insulin challenge was selected based on the established kinetics of insulin-stimulated AKT phosphorylation in HepG2 cells, where peak phosphorylation occurs at approximately 10–15 min after insulin stimulation [48,49,51,52]. After treatment, CTG reagent was added to wells containing cells under both conditions (with and without acute insulin pulse), and luminescence was measured to assess ATP-derived viability. Cell viability was expressed relative to the respective vehicle control to assess the capacity of the inhibitors to suppress both basal survival and the metabolic response to acute signaling activation.

### 2.6. Western Blot Analysis

For protein expression assessment, cells were lysed in RIPA buffer (ThermoFisher Scientific, Rockford, IL, USA, Cat. No. 89900) and protein concentrations were determined using BCA (ThermoFisher Scientific, IL, USA, Cat. No. 23227). The proteins were separated by SDS-PAGE on Bio-Rad Mini-PROTEAN TGX Precast Protein Gels at 10% acrymlamide (Bio-Rad, Hercules, CA, USA, Cat. No. 4561034), which provides extended electrophoretic resolution across the 20–200 kDa range and transferred to a PVDF membrane. Membranes were blocked with TBS buffer (50 mM Tris, pH 7.6, 150 mM NaCl, 0.5% Tween-20) containing 5% BSA, and probed overnight at 4 °C with different primary antibodies (1:1000 dilution) recognizing total and/or phosphorylated targets (AKT, p-AKT, mTOR, p-mTOR, AS160, p-AS160, p70S6K, p-p70S6K, ERK, p-ERK, IRS-1, p-IRS-1, Bcl-2, BAK, BID, Bax, PCNA, Ki-67, GLUT1, and GLUT2) and a loading control protein (GAPDH). After multiple washes with TBS buffer, blots were incubated with horseradish peroxidase (HRP)-conjugated secondary antibodies (1:5000 dilution) for 1 h at room temperature, and signals were detected using Clarity Western ECL substrate (Cat. No. 1705060, Bio-Rad, Hercules, CA, USA). Densitometric quantification of the protein bands was performed using ImageJ software (Version 1.54, National Institutes of Health, Bethesda, MD, USA; available at https://imagej.net/ij/), an open-source, Java-based image processing program [53].

### 2.7. Caspase-3 Activity Assay

Caspase-3 activity was measured using the Caspase-3 assay kit (MyBioSource, San Diego, CA, USA, Cat. No. MBS9718955) following the manufacturer’s instructions. Briefly, treated cells were lysed, then centrifuged at 600× *g* for 5 min at 4 °C, and washed twice with 1 mL PBS. After centrifugation, cells were resuspended in 50 µL working cell lysis buffer and incubated on ice for 15–20 min. Following incubation, cell lysates were centrifuged at 16,000× *g* for 5 min at 4 °C. Supernatants were then mixed with 50 µL of working reaction buffer and 5 µL of DEVD-pNA (4 mM) in a 96-well plate and incubated at 37 °C. Caspase-3 activity was detected at 405 nm using a microplate reader (Synergy H1, BioTek, Winooski, VT, USA). Raw signals were normalized to the corresponding protein of each sample.

### 2.8. Apoptosis Assessment Using Annexin V/Propidium Iodide Staining and Flow Cytometry

We assessed apoptosis using the Annexin Apoptosis Kit (Thermo Fisher Scientific, Waltham, MA, USA, Cat. No. V13241) according to the manufacturer’s protocol. Briefly, treated cells were harvested and washed with cold phosphate-buffered saline (PBS). Cells were then resuspended in the supplied binding buffer, stained with Annexin-V and PI (propidium iodide), and incubated for 15 min at room temperature. Following staining, samples were analyzed immediately (within 1 h) by flow cytometry (BD FACSMelody™ Cell Sorter, San Jose, CA, USA). Primary gating was performed using Forward Scatter Area (FSC-A) versus Side Scatter Area (SSC-A) to identify the singlet cell population and exclude debris. To eliminate spectral spillovers, fluorescence compensation was applied using single-stained controls for both PI and FITC channels. Quadrant gating was used to categorize cells as viable (Annexin V^−^/PI^−^), early apoptotic (Annexin V^+^/PI^−^), late apoptotic (Annexin V^+^/PI^+^), and necrotic (Annexin V^−^/PI^+^). At least three biological replicates were analyzed with 10,000 events acquired per sample. Data were analyzed using FlowJo v11.1 software (BD Life Sciences, Ashland, OR, USA), and percentages of apoptotic cells (early and late apoptosis) were reported as ±SEM.

### 2.9. Glucose Uptake Assay

Treated cells were seeded in a 96-well plate and incubated at 37 °C for 48 h. Glucose uptake under basal and insulin-stimulated conditions was measured using the Glucose Uptake-Glo Assay (Promega Corporation, Madison, WI, USA, Cat. No. J1341), according to the manufacturer’s protocol. Briefly, cells were washed with PBS and incubated with 2-deoxyglucose (2DG) substrate, after which the detection reagent containing 2-deoxyglucose-6-phosphate (2DG6P) was added to each well. Luminescence signals were recorded using the GloMax Explorer system (Promega Corporation, Madison, WI, USA). The 2DG uptake data are presented as a percentage relative to the vehicle control, as previously shown [54].

### 2.10. Glycogen Quantification Assay

Glycogen contents were quantified in HepG2 cell lines under basal and insulin-stimulated conditions using the Glycogen Assay from Sigma-Aldrich (Darmstadt, Germany, Cat. No. MAK465) in accordance with the manufacturer’s protocol. Briefly, treated cells were lysed and centrifuged at 14,000× *g* to remove debris, and 10 µL of clear supernatant was transferred into a 96-well plate. The resultant glycogen was detected at 570 nm using a microplate reader (Synergy H1, BioTek, Winooski, VT, USA). Protein lysates were quantified in parallel using BCA for data normalization. Glycogen content was measured by subtracting blank controls from all sample readings and normalizing to the sample protein concentration.

### 2.11. Molecular Docking Analysis

Three-dimensional crystal structures from the RCSB Protein Data Bank (PDB) [55] were retrieved for AKT1 (3O96) [56], and SLC2A1/GLUT1 (5EQG). Protein preparation, involving the removal of water molecules (unless involved in binding), removal of co-crystallized ligands, addition of missing hydrogen atoms, and energy minimization, was conducted through iGEMDock v2.1 software [57]. Ligands used in the study included MK-2206, BAY-876, and co-crystallized inhibitors (IQO and 5RE). The structures of the ligands were retrieved from the PubChem database [58] in “.sdf” format and converted to “.pdb” format using OpenBabel version 2.3 software [59]. Molecular docking was conducted using iGEMDOCK with the GEMDOCK scoring function and a population size of 200 and 70 generations. Ten docking poses were generated for IQO, MK-2206, and 5RE, whereas four poses were generated for BAY-876. The generated poses were ranked according to the total docking score, with the interaction-energy profile including van der Waals, hydrogen-bonding, and electrostatic contributions. The pose with the most favorable total docking score for each ligand was selected for detailed structural interaction analysis. Docking poses were ranked based on total binding energy. Docked complexes were analyzed using Discovery Studio Visualizer [v24.1.0.23298] and PyMOL Molecular Graphic System [v.2.5.5] for structural visualization. The docking protocol was further assessed by re-docking the native co-crystallized ligands IQO and 5RE into their corresponding binding sites and comparing the resulting poses with their crystallographic orientations by root-mean-square deviation (RMSD).

### 2.12. Statistical Analysis

Data are presented as mean ± SEM, and results were analyzed using one-way Analysis of Variance (ANOVA) followed by Tukey’s post hoc test for multiple comparisons among all treatment groups using GraphPad Prism v11 (Boston, MA, USA). *p* values less than 0.05 were considered statistically significant. Primary endpoints (IC_50_ determination, and CTG cell viability, proliferation, target engagement, caspase-3 activity, Annexin V/PI, glucose uptake, and glycogen quantification under both basal and acute insulin-challenged conditions) were performed with three independent biological replicates (*n* = 3), and Western blot analyses were performed with at least with two technical replicates per condition per biological experiment. The multi-model MTT SynergyFinder analysis matrix was performed with two independent biological experiments (*n* = 2). Exploratory Western blot analyses of downstream signaling, IRS-1, parallel MAPK pathway (Supplementary Figure S2), and Bcl-2 family regulators (Supplementary Figure S3) were performed with two independent biological replicates (*n* = 2) and are presented as directional observations.

## 3. Results

### 3.1. Basal IC_50_ Profiling Identifies a Highly Synergistic Combination of MK-2206 and BAY-876 for Downstream Signaling and Metabolic Studies

**Figure 1:**
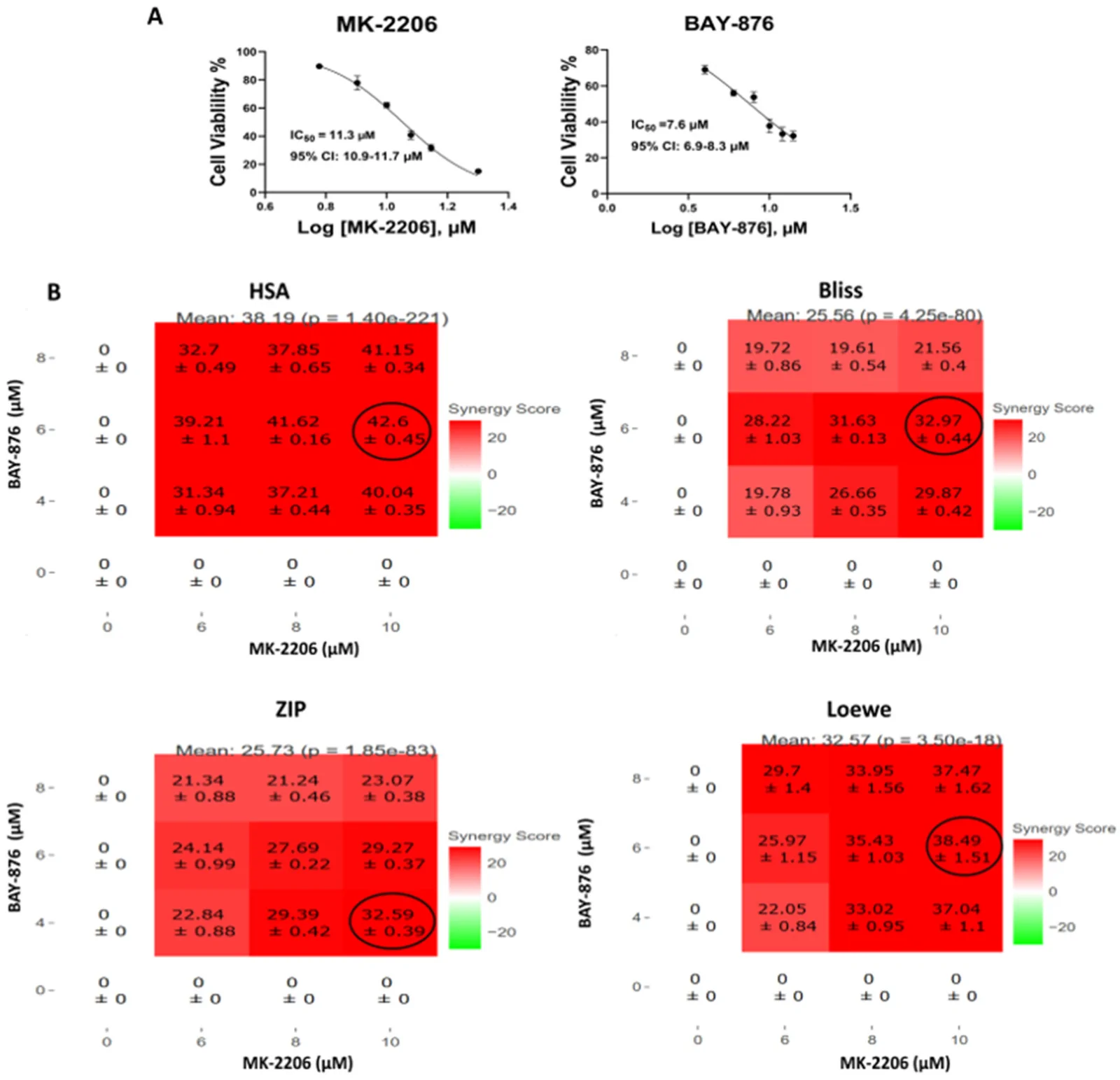
IC_50_ and Synergy Assessment for MK-2206 and BAY-876 in HepG2 Cells. (**A**) Concentration-response curves to determine the IC_50_ values for MK-2206 and BAY-876 in HepG2 cells under basal conditions (25 mM glucose). Cells were treated with a range of inhibitor concentrations for 48 h, and cell viability was assessed using the MTT assay. IC_50_ values were determined by nonlinear regression using a four-parameter logistic (4PL) sigmoidal model in GraphPad Prism v11. Solid lines represent the fitted sigmoidal curves. Data are presented as mean ± SEM (*n* = 3). MK-2206: IC_50_ = 11.3 µM (95% CI: 10.9–11.7 µM), R^2^ = 0.97; BAY-876: IC_50_ = 7.6 µM (95% CI: 6.9–8.3 µM), R^2^ = 0.87. (**B**) Multi-model synergy heat-maps from SynergyFinder analysis of HepG2 cell viability following 48 h treatment with the 3 × 3 concentration matrix of MK-2206 (6, 8, 10 µM) and BAY-876 (4, 6, 8 µM). Synergy scores are shown for four reference models: Highest Single Agent (HSA), Bliss independence (Bliss); Loewe additivity (Loewe); and Zero Interaction Potency (ZIP) from Experiment 1. Each heatmap represents the synergy score ± SEM for three technical replicates across concentration pairs for MK-2206 (x-axis) and BAY-876 (y-axis). Synergy scores > 10 indicate synergy, between −10 and 10 indicate additivity, and <−10 indicate antagonism. Black circles indicate the highest synergy score for each model.

First, we evaluated IC_50_ values for MK-2206 and BAY-876 under basal conditions in HepG2 cells. These experiments were conducted under hyperglycemic conditions (25 mM glucose) to determine IC_50_ values and identify synergistic dose combinations that remain effective despite high extracellular glucose availability for downstream mechanistic analyses (Figure 1A). For MK-2206, concentrations tested from 6–20 µM yielded an IC_50_ of 11.3 µM (95% CI: 10.9–11.7 µM, R^2^ = 0.97). For BAY-876, the IC_50_ was 7.6 µM (95% CI: 6.9–8.3 µM, R^2^ = 0.87) across 4–14 µM. Formal synergy analysis of MTT-derived cell viability using four independent reference models revealed the strongest interaction at 10 µM MK-2206 combined with 6 µM BAY-876. In Experiment 1, synergy scores were HSA = 42.6 ± 0.45, Bliss = 32.97 ± 0.44, Loewe = 38.49 ± 1.51, and ZIP = 29.27 ± 0.37. In Experiment 2, synergy scores were HSA = 53.14 ± 0.75, Bliss = 40.07 ± 0.85, Loewe = 49.3 ± 2.39, and ZIP = 36.15 ± 0.59 (Table 1; Figure 1B for Experiment 1 heatmaps; Supplementary Figure S1 for Experiment 2 heatmaps). All four scores in both biological experiments substantially exceeded the conventional synergy threshold of 10, providing convergent evidence of robust and reproducible pharmacological synergy between AKT and GLUT1 blockade across independent mathematical frameworks. Notably, formal synergy was observed across the entire 3x3 matrix, including at the lowest combination tested (6 µM MK-2206 + 4 µM BAY-876), which in Experiment 1 yielded synergy scores substantially exceeding the conventional threshold of 10 across all four reference models (HSA = 31.34 ± 0.94, Bliss = 19.78 ± 0.93, Loewe = 22.05 ± 0.84, and ZIP = 22.84 ± 0.88), with consistent synergy reproduced in Experiment 2, demonstrating that the synergistic interaction is not restricted to high-effect combinations. These results establish that the synergistic interaction between AKT and GLUT1 blockade is preserved in a hyperglycemic metabolic context, providing a quantitative framework for subsequent signaling and metabolic investigations.

### 3.2. Effect of MK-2206 and BAY-876 on AKT Phosphorylation and GLUT1 Expression

**Figure 2:**
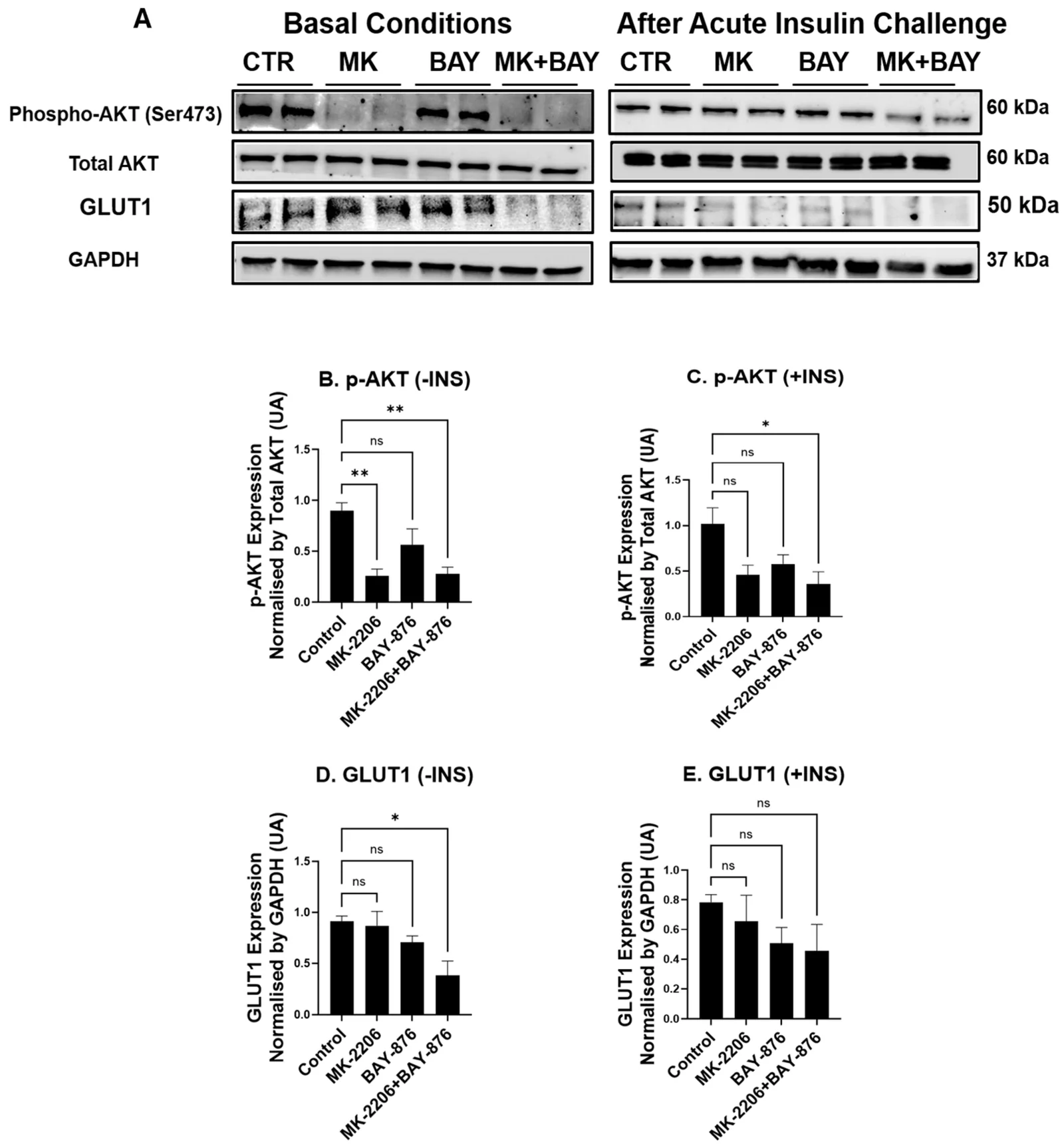
Effect of MK-2206 and BAY-876 on AKT phosphorylation and GLUT1 expression in HepG2 cells. (**A**) Representative Western blots showing bands of total AKT, phosphorylated AKT (Ser473), GLUT1, and GAPDH (loading control) across treatments and conditions. (**B**,**C**) Bar graphs representing the p-AKT/AKT ratio (Ser473 phosphorylation) under basal (-INS) and acute insulin-challenged conditions (+INS), shown as mean ± SEM (*n* = 3) for MK-2206 (10 µM), BAY-876 (6 µM), combination (MK-2206 + BAY-876), and vehicle control. (**D**,**E**) Bar graphs representing GLUT1 expression normalized to GAPDH under basal and acute insulin-challenged conditions, shown as mean ± SEM (*n* = 3) for the same treatments. Statistical comparisons were performed using one-way ANOVA with Tukey’s post hoc test. Asterisks denote significance (* *p* < 0.05, ** *p* < 0.01), and ns indicates no significance.

To characterize the pharmacological effects of MK-2206 (AKT inhibitor) and BAY-876 (GLUT1 inhibitor) in HepG2 cells, we assessed AKT phosphorylation (Ser473) and GLUT1 expression by Western blot analysis (Figure 2).

Under basal hyperglycemic conditions, MK-2206 alone significantly reduced the p-AKT expression (0.26 ± 0.06 vs. vehicle control 0.89 ± 0.08, *p* = 0.008), consistent with pharmacological inhibition of AKT phosphorylation. BAY-876 alone showed a directional reduction in p-AKT expression (0.56 ± 0.16), and the combination significantly reduced the p-AKT expression (0.28 ± 0.07, *p* = 0.009), producing an effect similar to MK-2206 alone (Figure 2B). Following the acute insulin challenge, MK-2206 alone showed a directional reduction in p-AKT expression (0.46 ± 0.12 vs. vehicle control 1.02 ± 0.18), with BAY-876 alone showing a similar directional reduction (0.58 ± 0.10). The combination treatment significantly reduced p-AKT expression (0.36 ± 0.13, *p* = 0.03), producing the largest effect among all treatments under acute insulin challenge (Figure 2C).

GLUT1 expression analysis revealed distinct treatment patterns. Under basal hyperglycemic conditions, MK-2206 alone showed minimal effect on GLUT1 expression (0.87 ± 0.14 vs. vehicle control 0.91 ± 0.05), consistent with the expected pharmacological selectivity of MK-2206 for AKT. BAY-876 alone showed a directional reduction in GLUT1 expression (0.7 ± 0.06), and the combination treatment significantly reduced GLUT1 expression (0.38 ± 0.14, *p* = 0.03; Figure 2D). Following the acute insulin challenge, all treatments produced directional reductions in GLUT1 expression compared to vehicle control (0.8 ± 0.04), with MK-2206 (0.66 ± 0.18), BAY-876 (0.51 ± 0.11), and the combination (0.44 ± 0.16) showing progressive reductions, though not reaching statistical significance (Figure 2E).

The significant reductions in p-AKT under MK-2206 and the combination are consistent with the pharmacological engagement of AKT signaling. The minimal effect of MK-2206 alone on GLUT1 expression under basal conditions supports the pharmacological selectivity of MK-2206 for AKT. The observed reductions in GLUT1 protein abundance under BAY-876 and combination treatment likely reflect compensatory or downstream cellular responses to BAY-876’s functional inhibition of GLUT1-mediated glucose transport rather than direct pharmacological modulation of GLUT1 expression. While several individual comparisons did not reach statistical significance, the consistent directional patterns and the significant findings for the primary target engagement comparisons provide a pharmacological context for interpreting the subsequent effects of these agents on metabolic, cell death, and proliferation endpoints.

### 3.3. MK-2206 and BAY-876 Combination Reduces Cell Viability and Proliferative Capacity

We evaluated the impact of single and combined inhibition of AKT and GLUT1 on HepG2 cell viability and proliferative capacity under basal hyperglycemic (25 mM glucose) and acute insulin-challenged conditions. The cell viability suppression effect of 10 µM MK-2206 combined with 6 µM BAY-876 identified in the MTT-based SynergyFinder analysis (Figure 1B) was further assessed under both basal and acute insulin-challenged conditions using the CellTiter-Glo (CTG) assay, which measures cellular ATP-derived content. Viability was assessed after 48 h of treatment with MK-2206 (AKT inhibitor) and BAY-876 (GLUT1 inhibitor). For the insulin-challenged group, cells received an acute 15 min insulin pulse after 48 h of treatment to assess the impact on maximal signaling activation.

**Figure 3:**
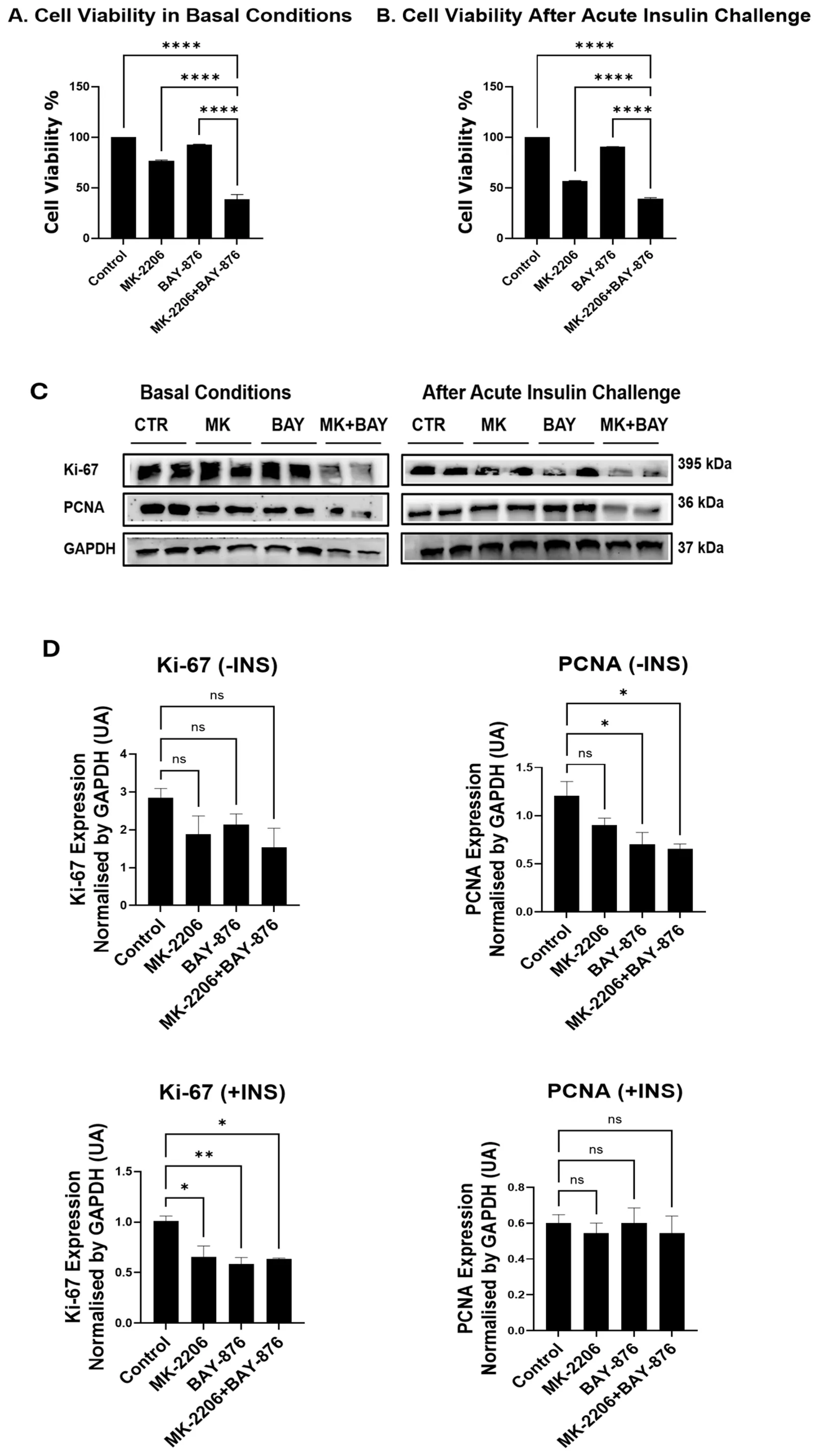
Effect of MK-2206, BAY-876, and their combination on cell viability and proliferation markers in HepG2 cells. (**A**,**B**) Bar graphs representing cell viability as a percentage relative to the respective vehicle control under basal and following acute insulin-challenge conditions, presented as mean ± SEM (*n* = 3) for MK-2206 (10 µM), BAY-876 (6 µM), combination (MK-2206 + BAY-876), and vehicle control. (**C**) Representative Western blots showing bands of Ki-67, PCNA, and GAPDH across treatments and conditions. (**D**) Densitometric quantification of Ki-67 and PCNA band intensities normalized to GAPDH, presented as relative expression to the respective vehicle control under basal (-INS) and following acute insulin-challenged conditions (+INS), shown as mean ± SEM (*n* = 3). Statistical comparisons were performed using one-way ANOVA with Tukey’s post hoc test. Asterisks denote significance (* *p* < 0.05, ** *p* < 0.01, **** *p* < 0.0001), and ns indicates no significance.

Inhibition of AKT (via MK-2206) and GLUT1 (via BAY-876) decreased cell viability, with the combination producing the greatest effects under basal hyperglycemic and acute insulin-challenged conditions. Under basal states, cell viability (measured via CTG) was reduced to 76.6% ± 0.77 by MK-2206 and to 92.67% ± 0.35 by BAY-876. Notably, the combination further reduced cell viability to 38.65% ± 4.76 (*p* < 0.0001 vs. both single agents; Figure 3A). Following the acute insulin challenge, MK-2206 reduced cell viability to 56.48% ± 0.6 and BAY-876 to 90.43% ± 0.24, whereas the combination achieved a markedly superior reduction to 39.46% ± 0.83 (*p* < 0.0001 vs. both single agents; Figure 3B).

Proliferation markers (Ki-67 and PCNA) were assessed by Western blot to further characterize the antiproliferative effects of dual AKT/GLUT1 inhibition (Figure 3C,D). Under basal hyperglycemic conditions, BAY-876 alone significantly reduced PCNA expression (0.70 ± 0.12 vs. vehicle control 1.21 ± 0.14, *p* = 0.04), and the combination similarly reduced PCNA expression (0.65 ± 0.05, *p* = 0.02), whereas MK-2206 alone showed minimal reduction compared to vehicle control (0.90 ± 0.07). Ki-67 expression under basal conditions showed directional reductions across treatments, with the combination producing the largest reduction (1.54 ± 0.50 vs. vehicle control 2.84 ± 0.24), though not reaching statistical significance. Following the acute insulin challenge, all three treatments significantly reduced Ki-67 expression compared to vehicle control (1.01 ± 0.05), with MK-2206 reduced to 0.65 ± 0.11 (*p* = 0.03), BAY-876 to 0.58 ± 0.06 (*p* = 0.01), and the combination to 0.63 ± 0.01 (*p* = 0.02). In contrast, PCNA expression under acute insulin challenge was similar across all treatments, with minimal reduction observed for MK-2206 and the combination (0.54 ± 0.05 and 0.54 ± 0.09, respectively, vs. vehicle control 0.59 ± 0.04), whereas BAY-876 alone showed no substantial change (0.60 ± 0.08) compared to control. These proliferation marker findings convergently support the antiproliferative effects observed in the CTG viability assay, with BAY-876-driven effects on PCNA under basal conditions and AKT-driven effects on Ki-67 under acute insulin-challenge conditions.

### 3.4. Dual Inhibition of AKT and GLUT1 Induces Cell Death in the Absence of Caspase-3 Activation

**Figure 4:**
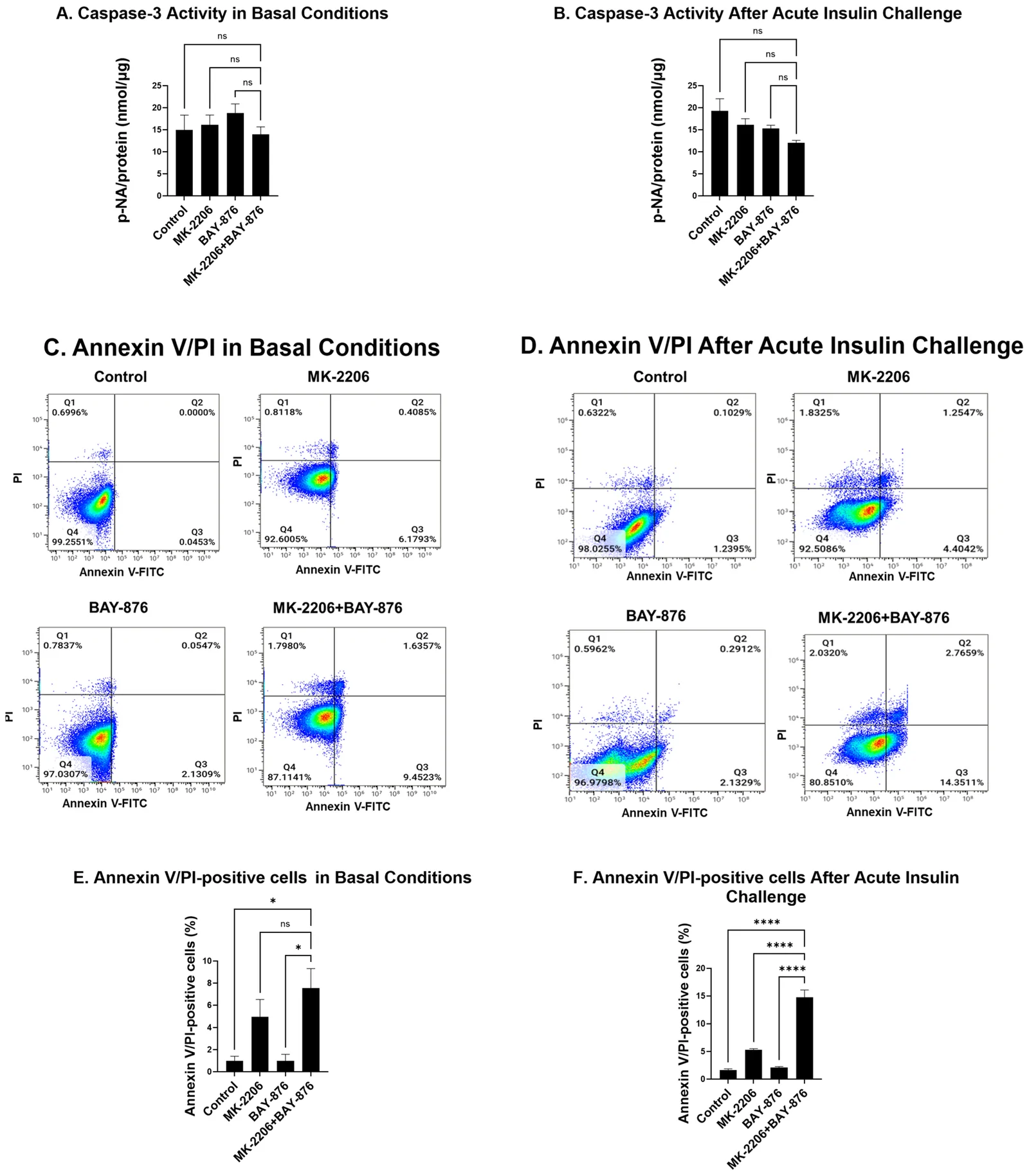
Cell death and caspase-3 activity in HepG2 cells under basal and acute insulin-challenged conditions. (**A**,**B**) Bar graphs representing caspase-3 activity p-NA/protein (nmol/µg) under basal and after acute insulin challenge conditions, shown as mean ± SEM (*n* = 3) for MK-2206 (10 µM), BAY-876 (6 µM), combination (MK-2206 + BAY-876), and vehicle control. (**C**,**D**) Representative dot plots for Annexin V-FITC/PI staining and flow cytometric analysis showing cells as viable (Q4: Annexin V^−^/PI^−^), early apoptotic (Q3: Annexin V^+^/PI^−^), late apoptotic (Q2: Annexin V^+^/PI^+^), and necrotic (Q1: Annexin V^−^/PI^+^) under basal and after acute insulin challenge conditions. (**E**,**F**) Bar graphs showing % of Annexin V-FITC/PI-positive cells (early + late) under basal and after acute insulin challenge conditions, shown as mean ± SEM (*n* = 3) for MK-2206 (10 µM), BAY-876 (6 µM), combination (MK-2206 + BAY-876), and vehicle control. Statistical comparisons were performed using one-way ANOVA with Tukey’s post hoc test. Asterisks denote significance (* *p* < 0.05, **** *p* < 0.0001), and ns indicates no significance.

To investigate the mechanism of cell death induced by AKT inhibition and GLUT1 blockade, we assessed cell death and executioner caspase activity under basal hyperglycemic and acute insulin-challenged conditions. The caspase-3 activity assay showed no induction under either basal or acute insulin-challenge conditions for single agents or the combination, indicating that the observed cell death occurs in the absence of caspase-3 activation (Figure 4A,B).

To quantitatively assess cell death, we performed Annexin V-FITC/PI flow cytometry and applied Tukey’s multiple comparison test to evaluate the superiority of the combination. Under basal hyperglycemic conditions, the combination of MK-2206 and BAY-876 increased the percentage of Annexin V-FITC/PI-positive cells (early and late) to 7.54% ± 1.77, significantly higher than control (0.993% ± 0.41, *p* = 0.02) and BAY-876 alone (0.996% ± 0.59, *p* = 0.024). This represented a non-significant numerical increase over MK-2206 alone (4.95% ± 1.58; *p* = 0.49; Figure 4C,D). However, this effect was markedly amplified following the acute insulin challenge. Under these conditions, the combination induced a pronounced increase in Annexin V-FITC/PI-positive cells (14.78% ± 1.31), significantly higher than both MK-2206 alone (5.33% ± 0.18, *p* < 0.0001) and BAY-876 alone (2.07% ± 0.19, *p* < 0.0001; Figure 4E,F). This condition-specific pattern indicates AKT-dominant effects under basal conditions, with combination-specific effects on cell death induction under acute insulin challenge. Collectively, these results suggest a shift from predominantly antiproliferative effects toward increased cell death in HepG2 hepatocyte-derived cells under dual AKT/GLUT1 inhibition, with increased Annexin V-FITC/PI-positive cell death occurring in the absence of detectable caspase-3 activation under the conditions and time point examined in this study.

### 3.5. Concurrent AKT Signaling Inhibition and GLUT1 Blockade Attenuates Metabolism in HepG2 Hepatocyte-Derived Cells

**Figure 5:**
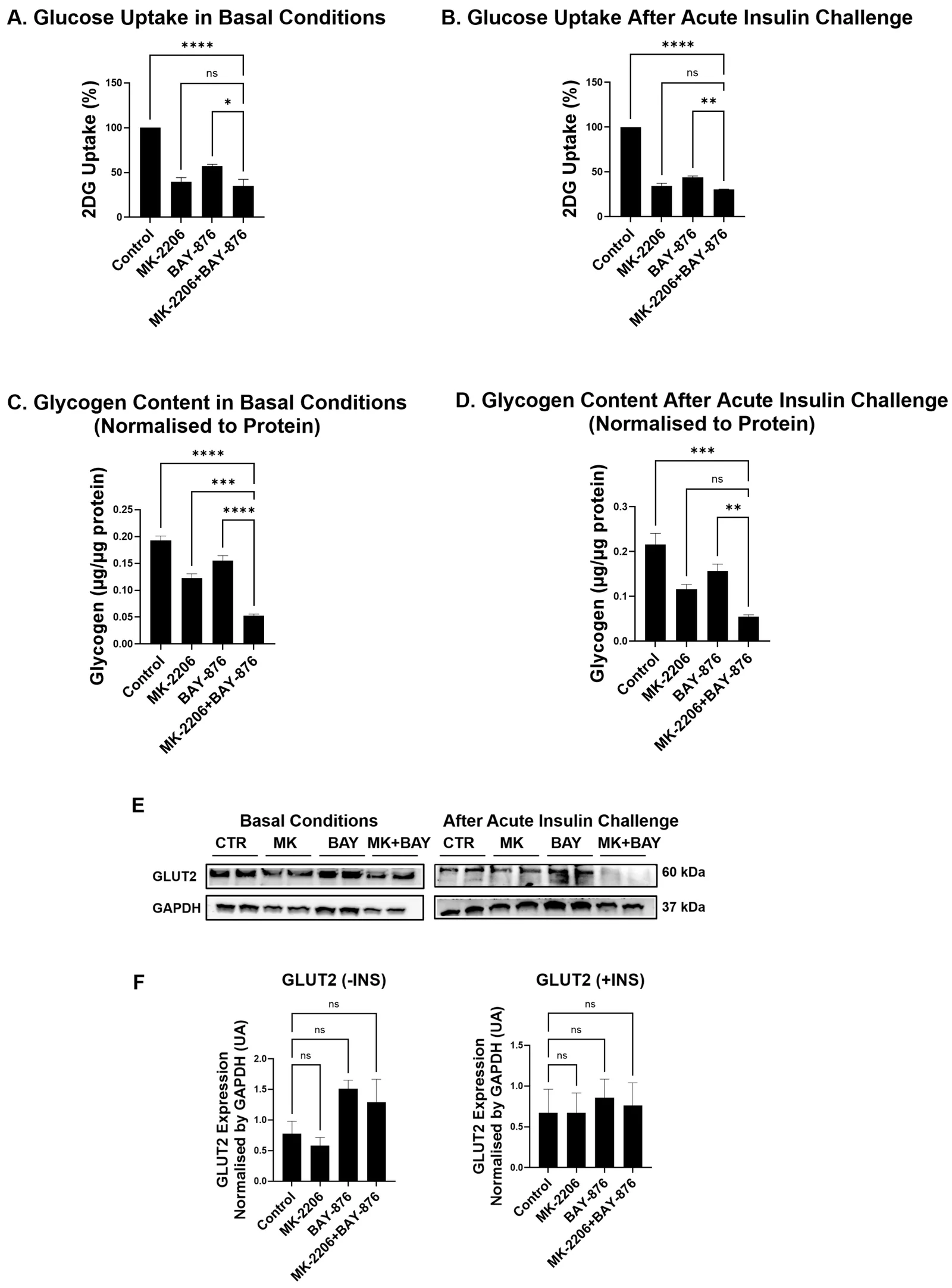
AKT signaling and GLUT1 blockade disrupt glucose uptake and glycogen storage with directional GLUT2 upregulation. (**A**,**B**) Bar graphs representing glucose uptake as a percentage relative to vehicle controls under basal and acute insulin-challenged conditions, shown as mean ± SEM (*n* = 3) for MK-2206 (10 µM), BAY-876 (6 µM), combination (MK-2206 + BAY-876), and vehicle control. (**C**,**D**) Bar graphs representing glycogen content normalized to protein (glycogen/µg protein) of the same samples under basal and acute insulin-challenged conditions, shown as a mean ± SEM (*n* = 3) for MK-2206 (10 µM), BAY-876 (6 µM), combination (MK-2206 + BAY-876), and vehicle control. (**E**) Representative Western blots showing GLUT2 and GAPDH bands across treatments and conditions. (**F**) Densitometric quantification of GLUT2 band intensities normalized to GAPDH, presented as relative expression, shown as mean ± SEM (*n* = 3). Statistical comparisons were performed using one-way ANOVA with Tukey’s post hoc test. Asterisks denote significance (* *p* < 0.05, ** *p* < 0.01, *** *p* < 0.001, **** *p* < 0.0001), and ns indicates no significance.

We examined how dual inhibition of AKT signaling and GLUT1-mediated glucose uptake disrupts cellular glucose utilization and glycogen storage under basal hyperglycemic and acute insulin-challenged conditions. AKT inhibition and GLUT1 blockade each reduced glucose uptake and glycogen content relative to vehicle controls, with the combination producing substantial reductions in both basal and insulin-stimulated conditions, showing endpoint-specific statistical patterns as detailed below (Figure 5).

Statistical analysis using one-way ANOVA with Tukey’s multiple comparisons revealed that under basal hyperglycemic conditions, the combination had a more pronounced effect by reducing glucose uptake to 35.13% ± 7.28 (*p* < 0.0001 vs. vehicle control). While this reduction was significantly greater than that achieved by BAY-876 alone (56.95% ± 2.24; *p* = 0.036), it was not significantly different from MK-2206 alone (39.57% ± 4.79; *p* = 0.89; Figure 5A). Similarly, following the acute insulin challenge, the combination reduced glucose uptake to 30.7% ± 0.24 (*p* < 0.0001 vs. vehicle control), showing significant superiority over BAY-876 alone (43.8% ± 1.76; *p* = 0.0021) but a non-significant decrease relative to MK-2206 alone (34.73% ± 2.74; *p* = 0.36; Figure 5B).

Glycogen content normalized to protein concentration mirrored these trends. Under basal hyperglycemic conditions, the combination reduced glycogen to 0.052 µg/µg protein ± 0.0028 (*p* < 0.0001 vs. vehicle control), which was significantly lower than both MK-2206 alone (0.123 µg/µg protein ± 0.008; *p* = 0.0009) and BAY-876 alone (0.155 µg/µg protein ± 0.009; *p* < 0.0001; Figure 5C). Following the acute insulin challenge, the combination reduced glycogen to 0.055 µg/µg protein ± 0.0038 (*p* = 0.0003 vs. vehicle control), representing a significantly greater reduction than BAY-876 alone (0.157 µg/µg protein ± 0.014; *p* = 0.0064) and a strong numerical trend over MK-2206 alone (0.116 µg/µg protein ± 0.01; *p* = 0.083; Figure 5D).

We further examined GLUT2 expression as a potential alternative glucose transporter that may compensate for GLUT1 inhibition. MK-2206 alone showed a modest directional reduction in GLUT2 expression under basal conditions (0.58 ± 0.13 vs. vehicle control, 0.77 ± 0.20) and no substantial change under acute insulin challenge (0.67 ± 0.24 vs. vehicle control, 0.67 ± 0.29). BAY-876 alone showed directional increases in GLUT2 expression under both basal (1.51 ± 0.14) and acute insulin-challenged conditions (0.86 ± 0.22). The combination treatment showed intermediate directional increases (basal: 1.29 ± 0.37 and acute insulin challenge: 0.76 ± 0.28). These directional increases in GLUT2 expression under BAY-876 and combination treatment did not reach statistical significance due to biological variability across independent experiments, but the consistent pattern across metabolic states suggests a potential compensatory pattern in response to GLUT1 inhibition (Figure 5E,F). Overall, these data reveal endpoint-specific patterns of pharmacological interaction between AKT signaling and GLUT1-mediated glucose transport blockade. Glucose uptake showed AKT-dominant effects (combination not statistically superior to MK-2206 alone under either condition), while glycogen content showed combination-specific effects under basal conditions and AKT-dominant effects under acute insulin challenge. Together, these findings indicate that AKT signaling and GLUT1 both support glucose utilization and glycogen storage in HepG2 hepatocyte-derived cells, with the directional GLUT2 pattern insufficient to prevent the substantial glucose uptake reduction observed under dual inhibition.

### 3.6. Exploratory Molecular Docking Provides Structural Context for MK-2206 and BAY-876 Interactions with Their Established Targets

Exploratory molecular docking was performed to assess whether MK-2206 and BAY-876 could be computationally accommodated within the established binding sites of AKT1 (PDB: 3O96) and GLUT1 (PDB: 5EQG), respectively. Re-docking of the co-crystallized ligands reproduced their crystallographic orientations, with RMSD values of 0.313 Å for IQO and 0.200 Å for 5RE. Among the generated poses, the selected MK-2206 and BAY-876 poses had docking scores of −113.983 and −118.188, respectively. Re-docking validates the reproduction of the crystallographic pose for these co-crystallized ligands rather than the poses predicted for MK-2206 and BAY-876. MK-2206 was positioned within the AKT1 allosteric pocket and showed predicted aromatic and hydrophobic contacts with surrounding residues, whereas BAY-876 was positioned within the inward-open GLUT1 cavity and showed predicted polar and hydrophobic contacts. The complete docking scores, residue-level interaction profiles, and structural visualizations are provided in Supplementary Tables S1 and S2 and Supplementary Figures S4 and S5. These docking findings provide exploratory structural context only and do not establish binding affinity, cellular target engagement, inhibition mechanism, or selectivity.

## 4. Discussion

This study provides mechanistic insight into how dual inhibition of AKT signaling and GLUT1-mediated glucose uptake shapes insulin-responsive glucose handling in HepG2 hepatocyte-derived cells. AKT and GLUT1 targeting has been previously investigated as a broader anti-tumor pharmacological strategy across multiple cancer contexts, including breast and lung cancer [26,27,29,30]. Our work reveals mechanistically distinct biological findings by characterizing this combination in a hepatocyte-derived model under both basal hyperglycemic and acute insulin-challenged conditions, integrating formal multi-model synergy quantification, metabolic readouts, cell death analysis, and exploratory molecular docking within a single experimental framework. The study design assessed the effects of MK-2206, an allosteric AKT inhibitor, and BAY-876, a selective GLUT1 inhibitor, individually and in combination, on target engagement, cell viability, proliferation, cell death, and metabolic functions in HepG2 cells. By evaluating dual AKT/GLUT1 inhibition under standardized high-glucose culture conditions (25 mM glucose), our design provides a controlled experimental framework that complements the insulin-challenged condition analysis and enables characterization of pharmacological effects across distinct metabolic states. Although GLUT1 is not directly insulin-responsive in HepG2 cells, insulin can still potentiate glucose utilization through AKT-dependent mechanisms that operate independently of GLUT1 translocation. In this context, GLUT1 would provide constitutive basal glucose uptake to support insulin-driven metabolic and biosynthetic demands. Consequently, inhibiting AKT suppresses insulin-driven growth signaling, whereas inhibiting GLUT1 restricts basal glucose supply. Our data show that dual inhibition of AKT signaling and GLUT1-mediated glucose uptake produces endpoint-specific patterns of pharmacological interaction affecting insulin-responsive signaling and metabolic outputs, with implications for hepatic metabolism. Across multiple readouts (cell viability, basal glycogen content, and Annexin V/PI-positive cell death under acute insulin challenge), dual inhibition produced combination-specific effects statistically superior to both single agents, while other readouts (glucose uptake under both conditions, and glycogen under acute insulin challenge) showed AKT-dominant patterns, supporting a model in which AKT signaling and GLUT1-targeted pharmacological effects contribute differentially to specific aspects of glucose handling in HepG2 hepatocyte-derived cells. Previous work has shown that AKT promotes GLUT1 translocation to the plasma membrane, thereby increasing glycolytic flux and promoting tumor growth, metastasis, and chemoresistance [60,61]. Furthermore, it has been established that insulin drives hepatic glucose uptake and glycogen storage via PI3K/AKT signaling, underscoring the role of GLUTs in glucose metabolism in hepatocytes [62,63,64]. Our findings extend this framework by directly demonstrating, within a single hepatocyte model, that simultaneous pharmacological targeting of AKT signaling and GLUT1-mediated glucose transport produces endpoint-specific patterns rather than uniform amplification of effects across the metabolic network. A key aspect of this investigation was the initial concentration-response and synergy assessments, which revealed distinct potencies but strong interactions between the two agents under hyperglycemic conditions. The concentration-response analysis of MK-2206 and BAY-876 under basal conditions revealed IC_50_ values of 11.3 µM and 7.6 µM, respectively, with BAY-876 being more potent (Figure 1A). Formal synergy analysis of MTT-derived cell viability using four independent reference models (HSA, Bliss, Loewe, and ZIP) identified strong multi-model interactions at 10 µM MK-2206 with 6 µM BAY-876, with all four scores substantially exceeding the conventional synergy threshold of 10 in two independent biological experiments (Figure 1B; Supplementary Figure S1). This convergent multi-model evidence establishes genuine pharmacological interaction beyond additive single-agent activity and provides a quantitative framework that remains robust under the standard high-glucose (25 mM) conditions used in this study. This enhanced cell viability suppression at the selected combination was further assessed using the ATP-derived CellTiter-Glo (CTG) viability measurement performed in three biological experiments using a methodologically distinct functional viability measurement (ATP content vs. mitochondrial reductase activity), though both endpoints remain dependent on cellular metabolism (detailed below in the cell viability discussion). The formal synergy quantification was performed on cell viability as the primary screening endpoint, with functional and metabolic readouts revealing endpoint-specific patterns of pharmacological interaction. The observed patterns include combination-specific effects for some endpoints, AKT-dominant patterns for other endpoints, and exploratory or directional observations for signaling components that did not reach statistical significance. The variability in interaction magnitude across endpoints is consistent with the multi-step nature of glucose handling in HepG2 hepatocyte-derived cells, where AKT signaling, GLUT1-mediated substrate flux, and downstream synthetic capacity contribute differentially to specific outcomes.

The analysis of MK-2206 and BAY-876 effects on AKT phosphorylation and GLUT1 expression in HepG2 cells provided a pharmacological context for interpreting the subsequent mechanistic analyses (Figure 2). MK-2206 alone produced a statistically significant reduction in p-AKT expression under basal conditions, consistent with the established allosteric AKT inhibition mechanism of this pharmacological agent, which binds outside the ATP-binding pocket of AKT to prevent its phosphorylation and activation [65]. This significant reduction in p-AKT is consistent with pharmacological engagement of the intended target and supports using MK-2206 as a pharmacological tool for AKT pathway analysis in HepG2 cells. The pharmacological selectivity of MK-2206 for AKT was consistent with the minimal effect of MK-2206 alone on GLUT1 expression under basal conditions, indicating that observed combination effects on GLUT1 may reflect the pharmacological effect of BAY-876 rather than off-target activity of MK-2206. BAY-876 alone produced directional reductions in GLUT1 expression, consistent with pharmacological GLUT1 inhibition, though not reaching statistical significance. This may reflect the pharmacological nature of the inhibition, which affects GLUT1 function without necessarily reducing protein levels, combined with biological variability inherent to Western blot quantification. Consequently, the observed changes in GLUT1 protein abundance may represent compensatory or downstream cellular responses to functional inhibition rather than direct transcriptional regulation. Notably, BAY-876 also produced directional reductions in p-AKT expression, which likely reflects indirect effects through glucose restriction-mediated metabolic stress rather than direct pharmacological effects of BAY-876 on AKT signaling. This observation is consistent with published literature on crosstalk between glucose sensing and PI3K/AKT signaling pathway, where reduced glucose availability can modulate AKT activation through altered insulin receptor signaling, AMPK activation, and metabolic stress response pathways [66]. This observation of indirect p-AKT modulation by BAY-876 is consistent with the interconnected nature of metabolic and signaling pathways [67,68]. The combination treatment produced significant reductions in p-AKT expression under both metabolic states and a significant reduction in GLUT1 expression under basal conditions. Directional reductions in GLUT1 expression under insulin conditions with the combination did not reach statistical significance, likely reflecting biological variability at *n* = 3. While the mixed significance pattern across conditions limits definitive quantitative conclusions for each comparison, the consistent directional patterns, combined with significant findings for the primary target engagement measures, provide pharmacological context that supports the interpretation of subsequent functional analyses. Exploratory characterization of downstream signaling components (AS160, mTOR, p70S6K), the upstream signaling regulator IRS-1, and the MAPK pathway (ERK1/2) (Supplementary Figure S2) provided directional observations consistent with the pharmacological effects observed in Figure 2.

ATP-derived viability measurements showed that inhibition of AKT (MK-2206) and GLUT1 (BAY-876) decreased cell viability, with the combination producing the greatest reduction across basal hyperglycemic and acute insulin-challenged states (Figure 3A,B). This combination-specific effect on cell viability was statistically superior to both agents under both metabolic conditions. Western blot analysis of proliferation markers revealed condition-specific patterns. Under basal hyperglycemic conditions, BAY-876 and the combination significantly reduced PCNA expression, with a comparable reduction between the two treatments, indicating a BAY-876-driven effect. Under acute insulin-challenged conditions, all three treatments significantly reduced Ki-67 expression with a comparable reduction, indicating a treatment-general effect rather than combination-specific suppression (Figure 3C,D). These condition-specific proliferation marker patterns, combined with the combination-specific cell viability effects, suggest that dual AKT/GLUT1 inhibition impairs cell survival through multiple context-dependent mechanisms. The convergent evidence from independent methodologies (ATP-derived viability and Western blot proliferation markers) supports the antiproliferative effect of dual AKT/GLUT1 inhibition, with the combination-specific effect on cell viability representing the strongest functional finding. These findings align with the parallel reductions in glucose uptake and glycogen storage, suggesting that dual AKT/GLUT1 inhibition disrupts signaling, cell survival, and proliferation, consistent with elevated metabolic stress under combined pathway inhibition.

Our results further demonstrate that dual AKT/GLUT1 inhibition shows a pattern suggesting a shift from predominantly antiproliferative effects toward increased cell death, more pronounced with the combination than with either agent alone. The Annexin V/PI flow cytometry analysis demonstrated increased Annexin V/PI-positive cells with combination treatment compared to control and BAY-876 alone under basal hyperglycemic conditions, with combination-specific increases observed following acute insulin challenge (Figure 4C,F). The insulin-induced cell death response is consistent with increased metabolic vulnerability under acute insulin-challenged conditions when signaling and metabolic demands are elevated. Notably, the observed increased cell death occurred in the absence of detectable caspase-3 activation under any treatment condition, contrasting with previous reports of caspase-3 activation following single-agent MK-2206 treatment in HepG2 cells [69] (Figure 4A,B). This dissociation between Annexin V/PI-positive cells and caspase-3 activity represents a phenotypic observation under combined pharmacological targeting of AKT and GLUT1 in a hepatocyte-derived model. This pattern establishes increased Annexin V/PI positivity without detectable caspase-3 activation under the conditions and time point examined in this study. Caspase activation may be transient or involve other caspases (e.g., caspase-7/8/9) not measured in this study, and a definitive mechanistic classification of the cell death phenotype would require additional characterization. Multiple alternative cell death mechanisms may be consistent with this pattern, including AIF translocation, calpain activation, cathepsin release, or necroptotic cell death signaling. Exploratory characterization of Bcl-2 family regulatory dynamics (Supplementary Figure S3) provided directional observations of condition-specific expression patterns supporting the cell death phenotype quantitatively established by Annexin V/PI. We acknowledge that the specific alternative cell death pathway responsible has not been directly characterized in this study. Definitive identification of the responsible mechanism, including measurement of AIF translocation, calpain activation, cathepsin release, necroptosis markers, and additional apoptotic regulators (Bim, cleaved PARP, and caspase-7/8/9), alongside comprehensive Bcl-2 family characterization, represents an important direction for future investigation. The insulin-enhanced cell death response suggests potential therapeutic relevance of dual AKT/GLUT1 targeting in the context of elevated insulin signaling, where cells may be particularly susceptible to the increased cell death response under heightened metabolic demand.

Our findings demonstrate that dual pharmacological targeting of AKT signaling (via MK-2206) and GLUT1-mediated glucose transport (via BAY-876) produces substantial effects on insulin-responsive metabolism in HepG2 hepatocyte-derived cells. This combination produced substantial reductions in glucose uptake and glycogen content under both basal hyperglycemic and acute insulin-challenged conditions, with endpoint-specific patterns discussed below (Figure 5). The observed reductions in glucose uptake should be interpreted in the context of the concurrent combination-specific reductions in cell viability observed under the combination treatment, as the total cellular uptake capacity per well reflects both functional transport activity and cell number (Figure 3). The concurrent combination-specific reductions in protein-normalized glycogen content provide complementary evidence for per-cell metabolic effects of dual AKT/GLUT1 inhibition that extend beyond the total cellular uptake per well measurements. The observed reductions in glucose uptake by BAY-876 and the combination are consistent with the reported pharmacological GLUT1 inhibition by BAY-876, though direct per-cell target engagement was not quantified in the present study (Figure 2). Notably, GLUT2 expression analysis revealed a distinct pattern, with BAY-876 alone and the combination producing directional increases in GLUT2 expression across both basal and acute insulin-challenged conditions, suggestive of a directional pattern that may reflect a compensatory response to pharmacological GLUT1 inhibition, though this interpretation remains exploratory in the absence of statistical significance and direct target engagement validation (Figure 5E,F). While GLUT2 is normally expressed in human liver cells to facilitate bidirectional glucose flux, hepatocyte-derived immortalized cell lines often show reduced GLUT2-mediated transport compared with primary hepatocytes [22,70,71]. The observed pattern is consistent with GLUT2’s established role as a major glucose transporter in hepatocytes. However, this directional upregulation appears functionally insufficient, as the substantial reductions in glucose uptake persisted despite the directional GLUT2 increases, suggesting that GLUT2 upregulation does not fully compensate for GLUT1 inhibition in maintaining glucose uptake. In contrast, MK-2206 alone showed minimal effects on GLUT2 expression, suggesting that AKT signaling may not substantially regulate GLUT2 in HepG2 cells under these conditions. This is consistent with published evidence identifying hepatocyte-specific transcription factors (HNF-1α, HNF-4α, and FOXA2) as key regulators of GLUT2 expression in hepatocytes [72]. Together, these observations are consistent with the interpretation that the observed directional GLUT2 upregulation may reflect a specific response to GLUT1 inhibition. While the current study utilizes the highly selective inhibitor BAY-876 to disrupt GLUT1 function, the observed metabolic shifts are consistent with those reported following genetic suppression of SLC2A1 in other hepatocyte-derived models [25]. These observations are consistent with the reported pharmacological selectivity of BAY-876 for GLUT1 over GLUT2, though the current study does not include surface GLUT1 quantification or genetic target validation (e.g., SLC2A1/GLUT1 knockdown) that would be required to conclusively demonstrate GLUT1-specific cellular target engagement [31]. Future investigations incorporating surface GLUT1 quantification and genetic silencing of SLC2A1 will provide direct validation of GLUT1-mediated on-target effects. Further glucose uptake measurements revealed substantial declines in basal uptake following treatment with MK-2206 or the combination, while BAY-876 alone produced a slightly smaller effect (Figure 5A). Under the acute insulin challenge, AKT blockade again exhibited a stronger inhibitory effect than GLUT1 inhibition alone, and dual targeting yielded the most pronounced suppression (Figure 5B). Although the combination showed statistical superiority over BAY-876 alone, the decrease relative to MK-2206 alone represented a strong but non-significant trend, suggesting that AKT signaling plays a dominant role in maintaining both basal and insulin-mediated substrate flux. Glycogen data mirrored these trends, with basal glycogen significantly reduced by the combination compared with either MK-2206 or BAY-876, whereas insulin-stimulated glycogen showed the numerically strongest suppression under AKT/GLUT1 co-inhibition (Figure 5C,D). This differential pattern between glucose uptake and glycogen synthesis reflects the multi-step nature of glucose handling in HepG2 hepatocyte-derived cells. Glucose uptake is primarily regulated by transporter trafficking through the AKT/AS160 axis, where AKT inhibition by MK-2206 had a dominant effect on this readout, and BAY-876-mediated GLUT1 blockade produces limited additional effect when AKT signaling is suppressed [73]. In contrast, glycogen synthesis requires both substrate availability (mediated by GLUT1) and synthetic capacity (regulated by the AKT/GSK-3β/glycogen synthase pathway). Dual inhibition is expected to affect both substrate supply and synthetic enzyme regulation. Under basal hyperglycemic conditions, this combined disruption produced an amplified effect on glycogen storage that significantly exceeded the effect of both single agents, consistent with the combination-specific suppression observed in this metabolic readout (Figure 5C). Under acute insulin-challenged conditions, the combination was significantly superior to BAY-876 alone and showed a strong but non-significant trend over MK-2206 alone, again consistent with the dominant role of AKT signaling in maintaining glucose handling in HepG2 hepatocyte-derived cells under insulin stimulation (Figure 5D). Our data are consistent with published evidence that GLUT1 contributes to glucose uptake in HepG2 cells alongside GLUT2, though the pharmacological effects observed here cannot definitively establish per-cell GLUT1 contribution without genetic target validation. While GLUT1 inhibitors limit uptake by reducing substrate flux, AKT inhibitors modulate signaling that regulates both transporter trafficking and glycogen synthesis [74,75,76,77]. These results are aligned with established evidence that the PI3K/AKT signaling pathway is a key metabolic regulator in the liver, governing both glucose transporter trafficking and glycogen synthesis [64,76,78]. Specifically, AKT promotes glycogen storage primarily by phosphorylating and inactivating GSK-3α/β, thereby relieving the inhibition of glycogen synthase (GS) [76,79]. For instance, Simioni et al. demonstrated that the allosteric inhibitor MK-2206 reduces the phosphorylation of AKT1 and its downstream targets, including GSK-3α/β in hepatocyte-derived models [40]. This inhibition can effectively disrupt the signaling pathway required for insulin-stimulated glucose handling [80]. Furthermore, Zhong et al. showed that GLUT1 inhibition via BAY-876 effectively blunts the expression of glycogen synthesis-related genes in HCC cells, indicating a role for GLUT1 in hepatic glucose metabolism [42]. Our findings extend this literature by providing a unified view in HepG2 cells, demonstrating that dual AKT/GLUT1 inhibition produces endpoint-specific effects on glucose handling in HepG2 hepatocyte-derived cells, consistent with the pharmacological synergy quantified in the viability analysis. HepG2 is among the most widely used hepatocyte-derived cell lines for investigating hepatocyte insulin signaling, glucose metabolism, and glycogen handling, owing to its retention of several key hepatocyte features including insulin responsiveness, expression of gluconeogenic enzymes, functional PI3K/AKT signaling, and the capacity to store glycogen [36,49,81]. The cell line has been extensively employed in in vitro experiments to dissect the IRS-1/PI3K/AKT/GSK-3β/glycogen synthase axis that governs hepatic glucose uptake and glycogen synthesis, making it particularly suited to the mechanistic questions addressed in the present study [36,49]. Although HepG2 has been reclassified as hepatoblastoma-derived rather than HCC, this reclassification does not diminish its established utility for investigating the integration of insulin signaling with glucose transporter function and glycogen metabolism in hepatocyte-derived cells, which is the central focus of this work [49,82]. HepG2 cells display constitutive activation of certain signaling nodes and quantitative differences from primary hepatocytes, which may influence the absolute magnitude of the responses observed but are consistent with the altered metabolic phenotype commonly reported in hepatocyte-derived immortalized lines [81]. Further studies in additional hepatocyte-derived models, such as HepaRG cells, primary hepatocytes, or in non-tumorigenic hepatocyte cell lines such as THLE-2, will help define the broader applicability of these mechanistic findings to hepatocyte glucose handling. Overall, these data reveal endpoint-specific patterns of pharmacological interaction, with combination-specific effects observed for basal glycogen and AKT-dominant effects characterizing glucose uptake under both metabolic states as well as acute insulin-challenged glycogen content. The consistent findings across BAY-876 and combination treatment showing directional GLUT2 upregulation suggest a potential compensatory response to GLUT1 inhibition, though these directional patterns did not reach statistical significance.

The exploratory docking analysis provided additional structural context for the established binding sites of MK-2206 and BAY-876. Both compounds can be computationally accommodated within their respective sites, with MK-2206 positioned in the AKT1 allosteric pocket and BAY-876 within the GLUT1 cavity, consistent with reported binding modes for these inhibitors. This docking analysis does not establish binding affinity, cellular target engagement, inhibition mechanism, or isoform selectivity, and is presented as structural context only.

We acknowledge several limitations of this study. The treatment duration (48 h drug exposure with a 15 min acute insulin challenge) was chosen to evaluate the integrated effects of dual AKT/GLUT1 inhibition on insulin-responsive signaling output at peak activation. The 48 h window provides sustained inhibition sufficient for secondary cellular responses (proliferation changes, apoptotic priming, and metabolic adaptation), while the 15 min insulin challenge captures the peak p-AKT activation kinetics observed in HepG2 cells. We acknowledge that a formal time-course analysis across multiple time points would provide additional resolution of the temporal dynamics underlying the observed effects, and such characterization is an important direction for future studies. Additionally, the pharmacological glucose and insulin concentrations used in this study do not model physiological insulin action or diabetic conditions. Investigation under physiological concentrations represents an important direction for future research to establish translational relevance. Furthermore, several methodological limitations should be considered when interpreting the GLUT1-related findings. Glucose uptake was measured as total cellular uptake per well relative to vehicle control and was not normalized to viable cell number or protein content. Additionally, surface GLUT1 quantification and genetic target validation were not performed. Consequently, the conclusions regarding GLUT1 engagement should be interpreted as pharmacological effects of BAY-876 on glucose uptake at the well level rather than as definitive demonstration of GLUT1-specific cellular target engagement per viable cell. Direct assessment of surface GLUT1 localization, SLC2A1 knockdown/rescue, and normalized glucose uptake per viable cell would provide additional mechanistic insights and represent important directions for future investigations. While Western blotting is inherently a semi-quantitative technique, some comparisons within the three independent biological replicates (*n* = 3) did not reach statistical significance due to biological variability. Accordingly, these results are presented as directional observations, with emphasis on the statistically significant primary target effects. Importantly, the biological relevance of the observed protein alterations was independently supported by complementary functional assays that directly assessed the consequences of the molecular changes. Moreover, the use of a single hepatocyte-derived model (HepG2) limits the generalizability of the findings. Validation in primary hepatocytes and additional hepatocyte-derived (HepaRG) cell lines would broaden the mechanistic insights reported here. Additionally, the molecular docking analysis is exploratory and was based on single static crystal structures with finite conformational sampling. Although re-docking closely reproduced the native ligand orientations, the predicted interactions provide complementary structural context and do not independently establish binding affinity, target engagement, selectivity, or cellular mechanism.

## 5. Conclusions

**Figure 6:**
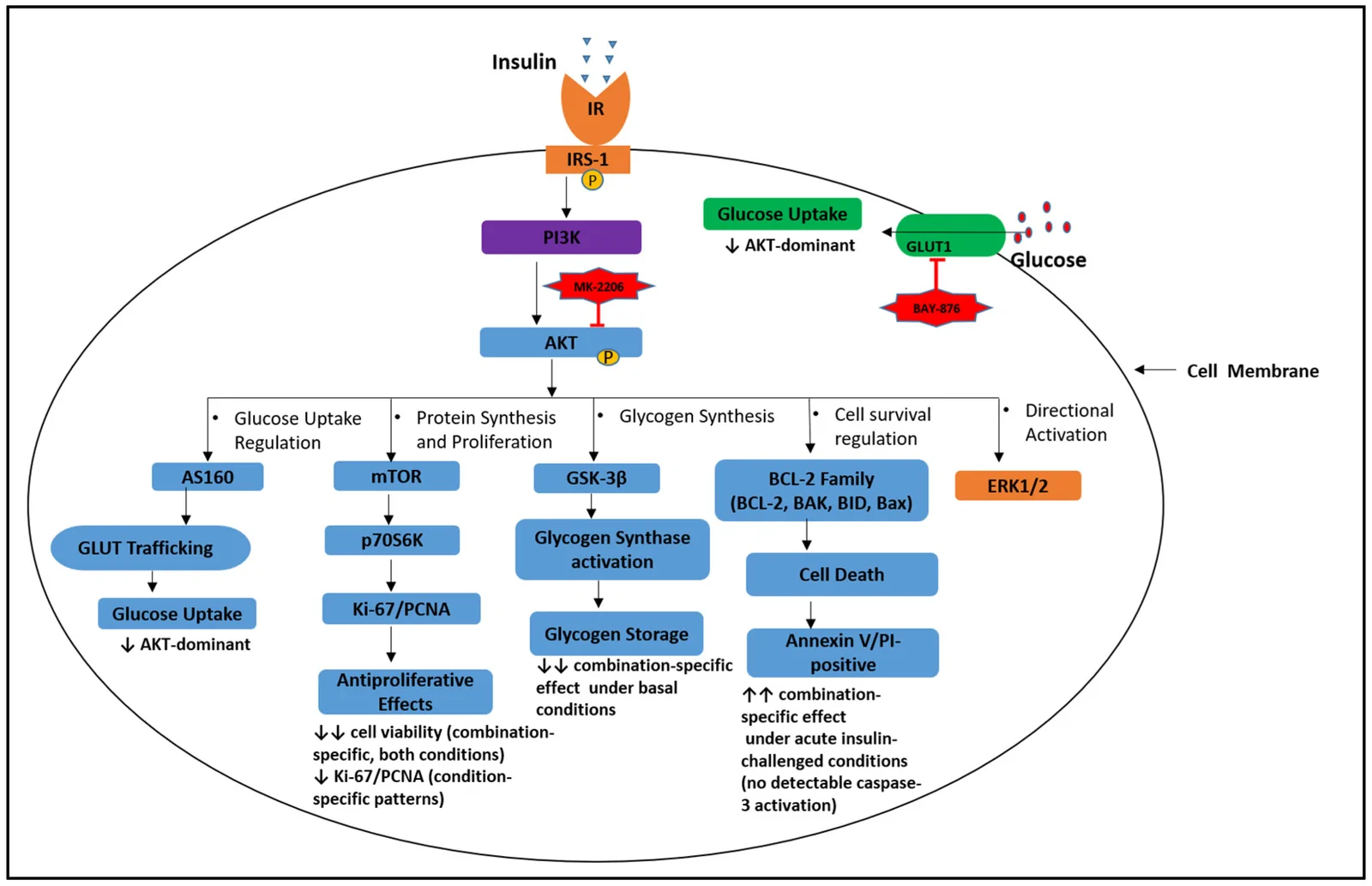
Integrated model of dual AKT and GLUT1 inhibition in HepG2 hepatocyte-derived cells. The schematic illustrates the concurrent effects of MK-2206 (an allosteric AKT inhibitor) and BAY-876 (a selective GLUT1 inhibitor) on insulin-responsive signaling, glucose metabolism, proliferation, and cell death. MK-2206 inhibits AKT activation suppressing downstream effectors AS160 (glucose transporter trafficking), mTOR/p70S6K (protein translation and proliferation), GSK-3β (glycogen synthase regulation), and BCL-2 family proteins (BCL-2, BAK, BID, Bax). BAY-876 directly inhibits GLUT1-mediated glucose transport at the cell membrane. Under dual inhibition, antiproliferative effects include combination-specific cell viability suppression under both basal and acute insulin-challenged conditions (↓↓), and condition-specific reductions in proliferation markers Ki-67 (across all treatments under acute insulin-challenged conditions) and PCNA (BAY-876 and combination treatments under basal conditions). Glucose uptake shows AKT-dominant regulation under both conditions (↓), glycogen storage shows combination-specific suppression under basal conditions (↓↓), and AKT-dominant effects under acute insulin challenge. Annexin V/PI-positive cell death shows combination-specific increases under acute insulin-challenged conditions in the absence of detectable caspase-3 activation (↑↑). Directional ERK1/2 activation from exploratory downstream signaling analyses (Supplementary Figure S2) is also depicted. Additional exploratory findings, including IRS-1 expression patterns, are reported in Supplementary Figure S2. AKT pathway components are shown in blue, GLUT1 pathway in green, and drug inhibition points in red.

Our data support a model in which combined pharmacological targeting of AKT signaling and GLUT1-mediated glucose uptake produces endpoint-specific patterns of pharmacological interaction on insulin-responsive metabolism, proliferation, and cell death in HepG2 hepatocyte-derived cells (Figure 6). Multi-model SynergyFinder analysis of MTT-derived cell viability demonstrated formal pharmacological synergy between MK-2206 and BAY-876, while functional endpoint analysis revealed endpoint-specific patterns: combination-specific suppression of cell viability under both basal hyperglycemic and acute insulin-challenged conditions, combination-specific reduction in glycogen storage under basal hyperglycemic conditions, AKT-dominant regulation of glucose uptake under both conditions and glycogen content under acute insulin challenge, and combination-specific induction of Annexin V/PI-positive cell death under acute insulin-challenged conditions in the absence of detectable caspase-3 activation. Additionally, proliferation markers showed condition-specific reduction patterns. Exploratory molecular docking analyses generated computationally predicted binding poses of MK-2206 at the AKT1 allosteric site and of BAY-876 with the GLUT1 transmembrane cavity, consistent with the established binding modes of these inhibitors.

Collectively, these findings characterize the pharmacological interaction between AKT-dependent signaling and BAY-876-mediated GLUT1 inhibition in HepG2 hepatocyte-derived cells. Our results support an endpoint-specific interpretation framework in which these signaling and substrate flux nodes differentially contribute to glucose handling, proliferation, and cell death in HepG2 cells. Combined pharmacological targeting of these pathways may serve as a useful mechanistic tool for dissecting insulin-responsive metabolism in hepatocyte-derived cells. Future studies extending these findings to additional hepatocyte-derived models, characterizing the specific cell death pathway engaged in the absence of caspase-3 activation, and integrating time-course analyses of the temporal dynamics of the observed effects will further refine the mechanistic understanding of dual AKT/GLUT1 inhibition in hepatic biology.

## Figures and Tables

**Table 1:** Multi-model synergy score for the 10 µM MK-2206 + 6 µM BAY-876 combination across two independent biological experiments.

Reference Model	Experiment 1 (Mean ± SEM)	Experiment 2 (Mean ± SEM)	Threshold
HSA	42.6 ± 0.45	53.14 ± 0.75	>10
Bliss	32.97 ± 0.44	40.07 ± 0.85	>10
Loewe	38.49 ± 1.51	49.3 ± 2.39	>10
ZIP	29.27 ± 0.37	36.15 ± 0.59	>10

## Data Availability

Data presented in this study are available in the article.

## Supplementary Materials

The following supporting information can be downloaded at: https://www.mdpi.com/article/10.3390/biomedicines14092133/s1, Figure S1: Multi-model synergy heatmaps from Experiment 2 demonstrate reproducibility of the synergistic interaction; Figure S2: Exploratory characterization of downstream signaling components, upstream regulator, and the parallel pathway under MK-2206 and BAY-876 treatment in HepG2 cells; Figure S3: Exploratory characterization of Bcl-2 family under MK-2206 and BAY-876 treatment in HepG2 cells; Figure S4: Validation of the molecular docking protocol by re-docking of native co-crystallized ligands; Figure S5: Structural visualization of the intra-molecular interactions of the ligands with the binding pocket of the target proteins. Table S1: Interaction-energy profiles of the generated docking poses for the co-crystallized reference ligands and investigated inhibitors within AKT1 (PDB ID: 3O96) and GLUT1 (PDB ID: 5EQG); Table S2: Intramolecular interactions of the ligands (MK-2206 and BAY-876) and co-crystalized inhibitors in the binding pocket of the target proteins (AKT1 and GLUT1).

## Abbreviations

The following abbreviations are used in this manuscript:

2DG	2-deoxyglucose
2DG6P	2-deoxyglucose-6-phosphate
AKT	Protein Kinase B
AS160	AKT substrate of 160 kDa
BAK	Bcl-2 antagonist/killer
Bax	Bcl-2-associated X protein
BCL2	B-cell Lymphoma 2
BID	BH3-interacting domain death agonist
Bim	Bcl-2 interacting mediator of cell death
Bliss	Bliss independence
CTG	CellTiter-Glo
DMSO	Dimethyl Sulfoxide
ERK1/2	Extracellular signal-regulated kinase 1 and 2
FITC	Fluorescein
FOXA2	Forkhead box A2
FOXO3a	Forkhead box O3a
GAPDH	Glyceraldehyde-3-phosphate dehydrogenase
GLUT	Glucose Transporter
GLUT1	Glucose Transporter 1
GLUT2	Glucose Transporter 2
GSK-3	Glycogen synthase kinase 3
HCC	Hepatocellular Carcinoma
HepG2	Human hepatoblastoma
HNF-1α	Hepatocyte nuclear factors 1α
HNF-4α	Hepatocyte nuclear factors 4α
HSA	Highest Single Agent
INS	Insulin
IR	Insulin resistance
IRS-1	Insulin receptor substrate-1
Ki-67	Marker of cellular proliferation
Loewe	loewe additivity
MAPK	Mitogen-activated protein kinase
mTOR	Mammalian target of rapamycin
MTT	3-(4,5-dimethyl-2-thiazolyl)-2,5-diphenyl-tetrazolium bromide
p53	Tumor Protein 53
p70S6K	70 kDa ribosomal protein S6 kinase
PCNA	Proliferating Cell Nuclear Antigen
PDB	Protein Data Bank
PI	Propidium Iodide
PI3K	Phosphatidylinositol-3-kinase
PVDF	Polyvinylidene fluoride
RCSB	Research Collaboratory for Structural Bioinformatics
siRNA	Small interfering RNA
SLC2A1	Salute Carrier Family 2 Member 1
ZIP	Zero interaction potency
