# Supplementary material for: Concurrent Inhibition of AKT and GLUT1 Reveals Endpoint-Specific Effects on Glucose Metabolism and Cell Death in HepG2 Hepatocyte-Derived Cells
Source: Biomedicines. 2026 Sep 21;14(9):2133. doi: 10.3390/biomedicines14092133 (PMC13604054; doi:10.3390/biomedicines14092133)
Supplement: Supplementary file 1 [file biomedicines-14-02133-s001.zip › biomedicines-4485297-supplementary.pdf]

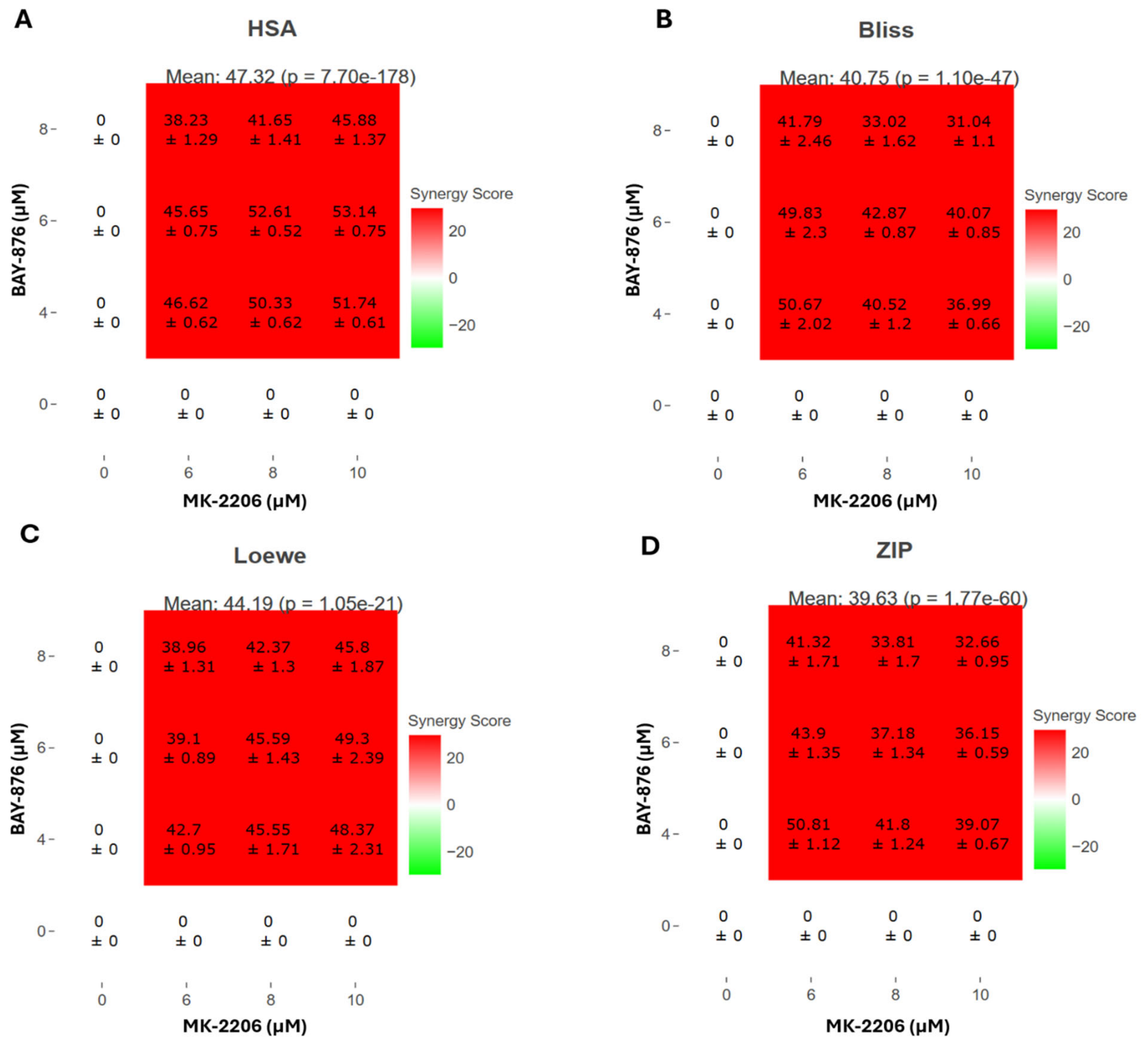

Supplementary Figure S1. Multi-model synergy heatmaps from Experiment 2 demonstrate reproducibility of the synergistic interaction. Synergy score heatmaps from SynergyFinder analysis of HepG2 cell viability following 48-hor treatment with the 3x3 concentration matrix of MK-2206 (6,8, 8 µM) and BAY-876 (4,6, 8 µM). Synergy scores are shown for four reference models in the second independent biological experiment: (A) Highest Single Agent (HSA); (B) Bliss independence (Bliss); (C) Loewe additivity (Loewe); and (D) Zero Interaction Potency (ZIP). Synergy scores > 10 indicate synergy, between -10 and 10 indicate additivity, and < -10 indicate antagonism. The synergistic interaction pattern across the matrix observed in Experiment 1 (Figure 1B) was reproduced in Experiment 2, with synergy scores substantially exceeding the conventional threshold of +10 across all four reference models throughout the matrix, demonstrating robust reproducibility of the pharmacological synergy between MK-2206 and BAY-876. Data represent mean of ± SEM of three technical replicates.

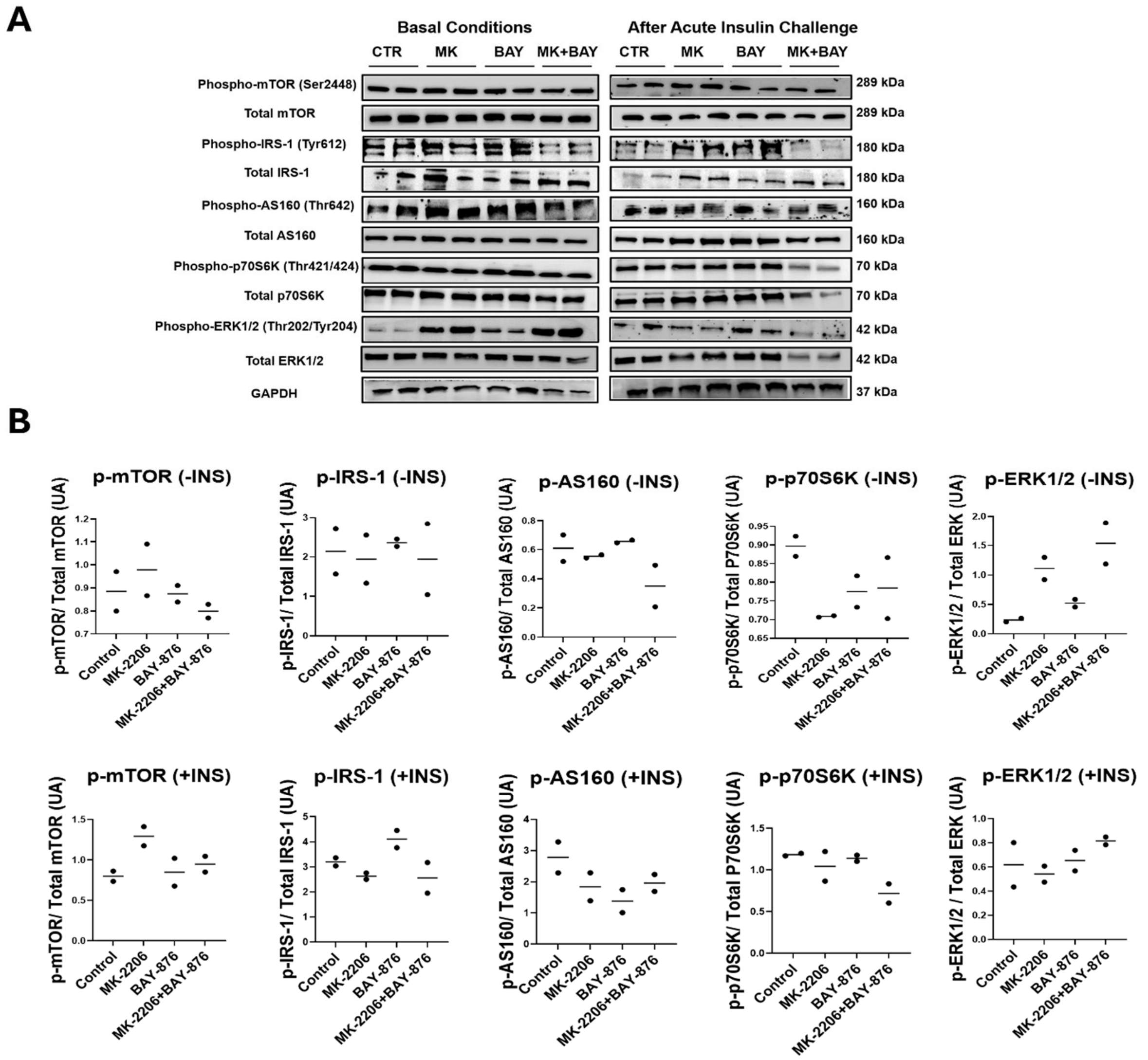

Supplementary Figure S2. Exploratory characterization of downstream signaling components, upstream regulator, and the parallel pathway under MK-2206 and BAY-876 treatment in HepG2 cells. (A) Representative Western blots showing the effects of MK-2206, BAY-876, and their combination on mTOR, p-mTOR, IRS-1, p-IRS-1, AS160, p-AS160, p70S6K, p-p70S6K, ERK1/2, and p-ERK1/2, with GAPDH as loading control in HepG2 cells under basal (-INS) and acute insulin challenge (+INS) conditions. Treatments: MK-2206 (10 µM), BAY-876 (6 µM), combination (MK-2206+BAY-876), and vehicle control. (**B**) Quantification of band densities from panel A, normalized to the corresponding total protein and plotted as mean of two replicates. This exploratory characterization was conducted with two independent experiments (n = 2) to provide directional support for the pharmacological effects on AKT phosphorylation and GLUT1 expression established in Figure 2.

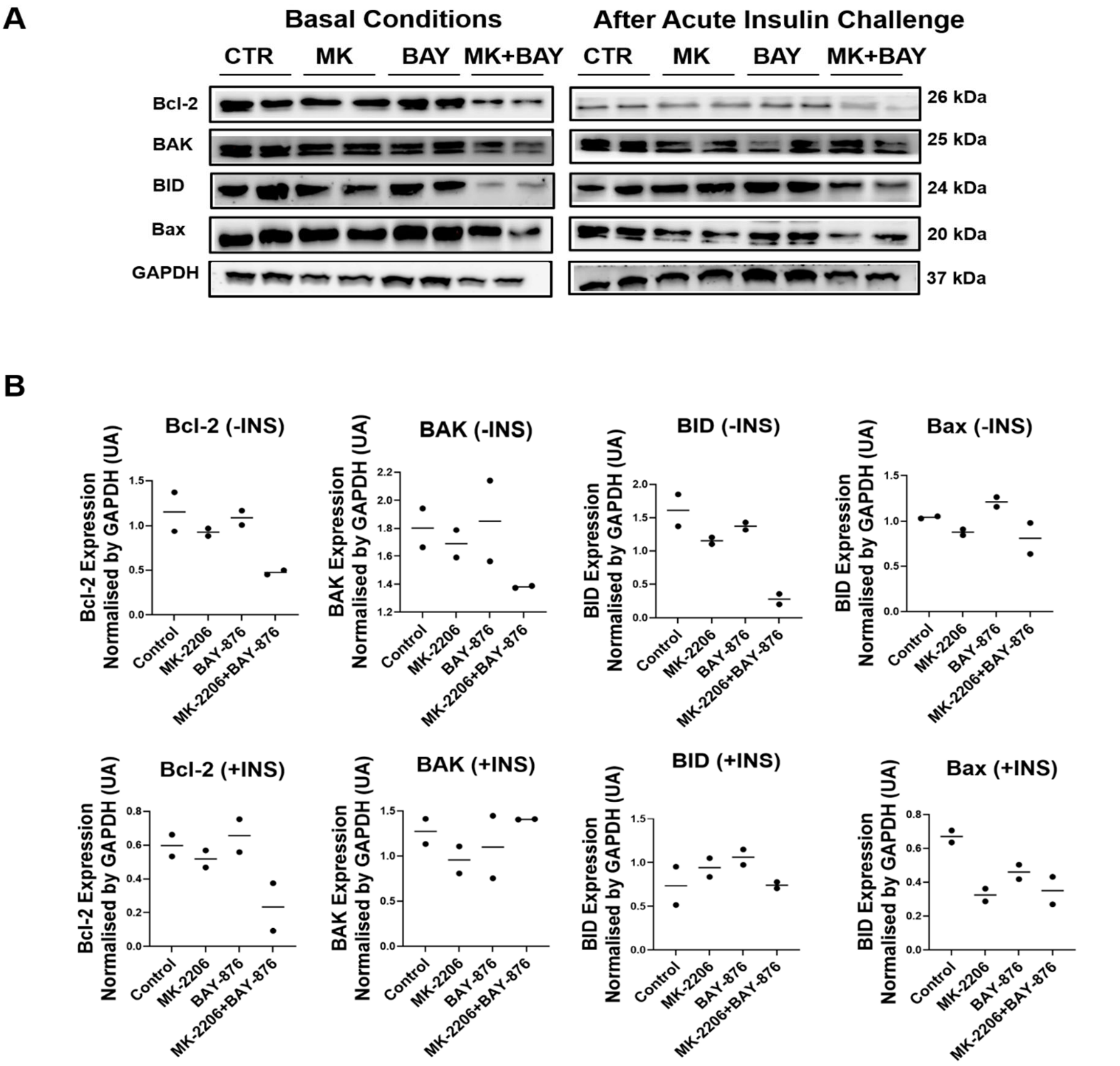

Supplementary Figure S3. Exploratory characterization of Bcl-2 family under MK-2206 and BAY-876 treatment in HepG2 cells. (A) Representative Western blots showing the effects of MK-2206, BAY-876, and their combination on Bcl-2 family regulatory proteins (BCL-2, BAK, BID, and Bax) with GAPDH as loading control in HepG2 cells under basal (-INS) and acute insulin challenge (+INS) conditions. Treatments: MK-2206 (10 μM), BAY-876 (6 μM), combination (MK-2206+BAY-876), and vehicle control. (**B**) Quantification of band densities from panel A, normalized to GAPDH and plotted as mean of two replicates. This exploratory characterization was conducted with two independent experiments (n = 2) to provide directional context for the apoptotic phenotype quantitatively established by Annexin V/PI flow cytometry in Figure 4.

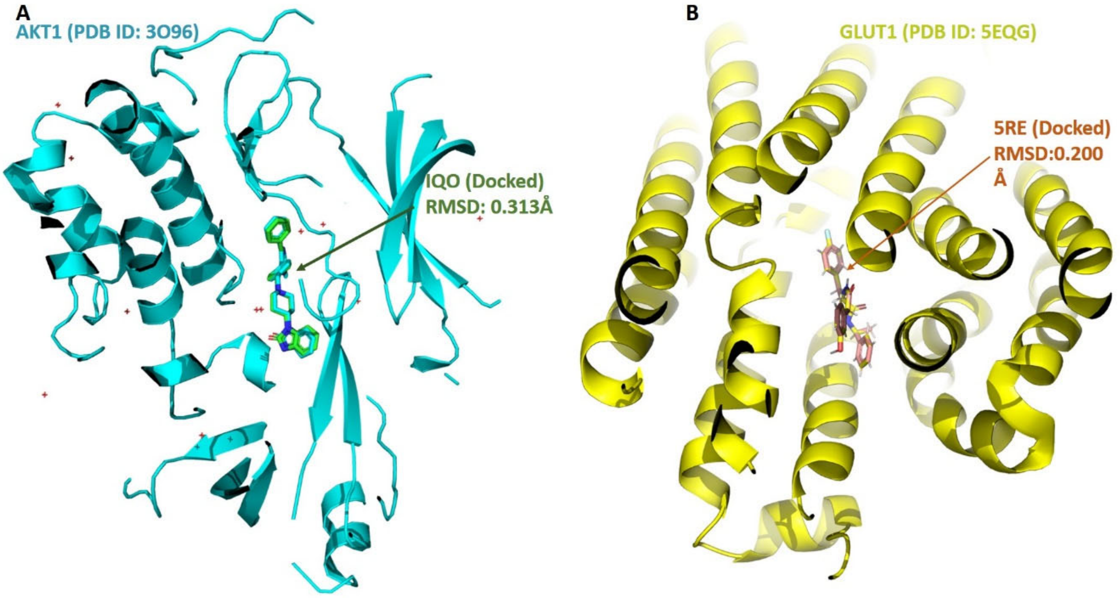

Supplementary Figure S4. Validation of the molecular docking protocol by re-docking of native co-crystallized ligands. Superimposition of the crystallographic and re-docked poses of (A) IQO within the AKT1 binding site (PDB ID: 3O96), yielding an RMSD of 0.313 Å, and (B) 5RE within the GLUT1 binding site (PDB ID: 5EQG), yielding an RMSD of 0.200 Å. The low RMSD values indicate close geometric agreement between the crystallographic and re-docked ligand orientations.

Supplementary Table S1: Interaction-energy profiles of the generated docking poses for the co-crystallized reference ligands and investigated inhibitors within AKT1 (PDB ID: 3O96) and GLUT1 (PDB ID: 5EQG).

Target protein	Ligand	Pose	Total docking	VDW	H-bond
AKT1 (3O96)	IQO	0	−105.964	−97.970	−7.994
		1	−163.360	−157.360	−6.000
		2	−121.147	−108.983	−12.164
		3	−121.143	−109.011	−12.132
		4	−126.718	−109.852	−16.866
		5	−135.814	−123.644	−12.170
		6	−121.141	−108.992	−12.149
		7	−115.362	−108.925	−6.437
		8	−121.131	−108.977	−12.154
		9	−163.394	−157.394	−6.000
	MK-2206	0	−110.237	−110.237	0
		1	−113.850	−113.850	0
		2	−113.919	−113.919	0
		3	−107.541	−107.541	0
		4	−104.704	−104.704	0
		5	−113.920	−113.920	0
		6	−113.983	−113.983	0
		7	−110.231	−110.231	0
		8	−102.119	−102.119	0
		9	−107.576	−107.576	0
GLUT1 (5EQG)	5RE	0	−165.884	−165.884	0
		1	−196.757	−195.445	−1.312
		2	−182.045	−174.801	−7.244
		3	−172.088	−169.588	−2.500
		4	−196.680	−195.712	−0.968
		5	−172.126	−169.626	−2.500
		6	−196.693	−195.489	−1.204
		7	−164.701	−162.201	−2.500
		8	−172.140	−169.640	−2.500
		9	−182.105	−174.899	−7.206
	BAY-876	0	−113.371	−113.371	0
		1	−113.416	−113.416	0
		2	−118.167	−118.167	0
		3	−118.188	−118.188	0

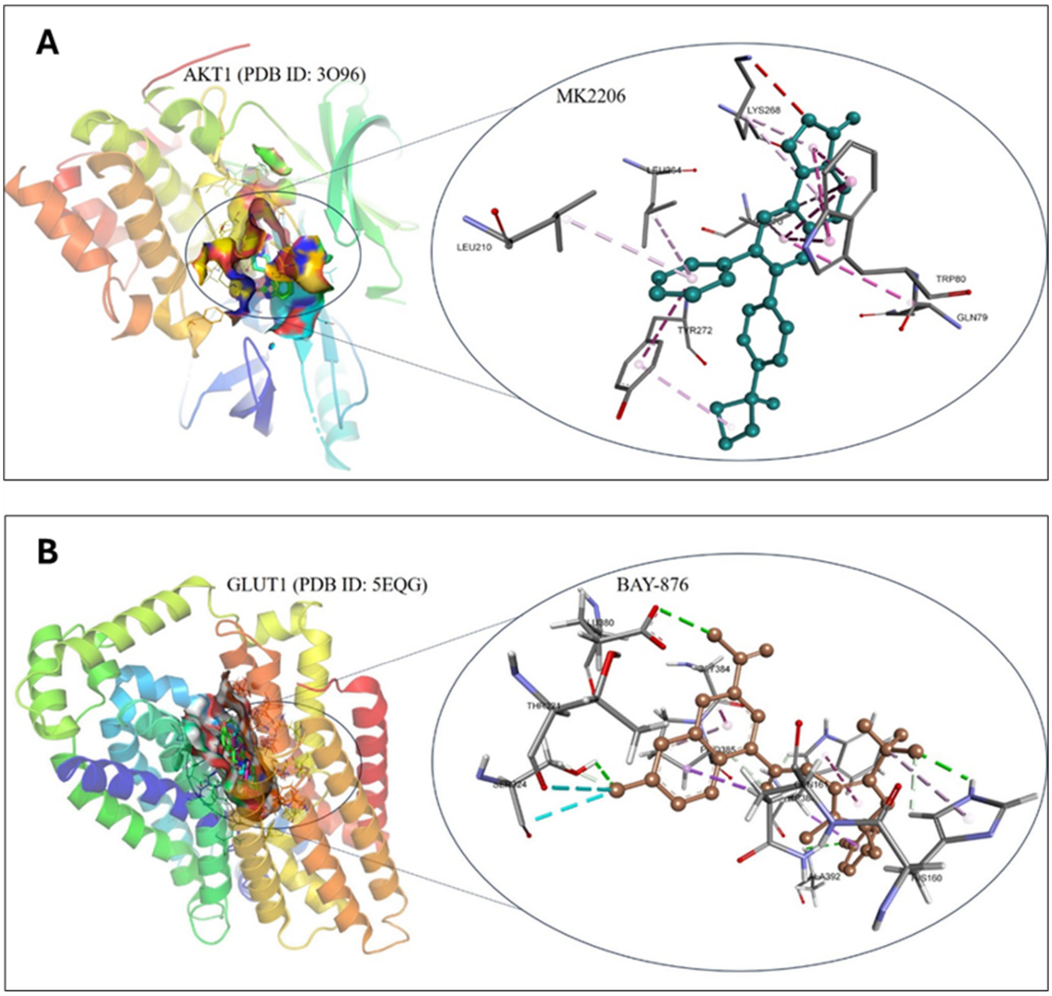

Supplementary Figure S5: Structural visualization of the intra-molecular interactions of the ligands with the binding pocket of the target proteins; (A) superimposed docking pose of the co-crystallized inhibitor-IQO, and the ligand-MK-2206 (shown in teal color ball and sticks) in the binding pocket of AKT shown in cartoon, (B) superimposed docking pose of the co-crystallized inhibitor-5RE, and the ligand-BAY-876 (shown in orange color ball and sticks) in the binding pocket of GLUT1 shown in cartoon. The figures are prepared in PyMol visualizer.

Supplementary Table S2: Intramolecular interactions of the ligands (MK-2206 and BAY-876) and co-crystalized inhibitors in the binding pocket of the target proteins (AKT1 and GLUT1).

Predicted interactions are reported for the selected docking poses of MK-2206 and BAY-876 and for the selected re-docked poses of the corresponding co-crystallized ligands, IQO and 5RE. Residue identities, interaction categories, and geometric distances were determined using Discovery Studio Visualizer v24.1.0.23298. The reported contacts represent geometry-based computational assignments.

Ligand	Interacting Residues	Distance (Å)	Interaction Type
IQO	HOH455	3.00	Water-mediated H-bond; Conventional H-bond
	SER205	2.60	Conventional H-bond
	ARG273	4.42	π–Cation
	ASP292	4.80	π–Anion
	ILE84	3.35	π–Sigma
	ILE84	3.76	
	LEU264	3.97	
	TRP80	3.74	π–π Stacked
	TRP80	4.04	
	TRP80	5.29	
	TRP80	4.66	
	TRP80	3.77	
	TRP80	4.12	
	VAL270	5.33	π–Alkyl
	LYS268	4.24	
	VAL270	5.31	
	LYS268	3.66	
	LEU210	4.25	
MK-2206	TRP80	3.73	π–π Stacked
	TRP80	5.19	
	TRP80	4.58	
	TRP80	4.62	
	TRP80	4.42	
	TRP80	4.19	
	TYR272	5.96	
	GLN79, TRP80	5.48	Amide–π Stacked
	TYR272	4.69	π–Alkyl
	VAL270	4.80	
	LYS268	3.63	
	LYS268	4.90	
	VAL270	4.59	
	LEU210	4.79	

	LEU264	5.25	
5RE	GLU380	2.66863	Conventional H-bond
	GLU380	3.60708	Halogen (Fluorine)
	PHE291	4.71429	π–π Stacked
	PHE26	4.42054	π–Alkyl
	TRP412	4.07172	
	TRP412	3.58238	
	ILE164	4.6218	
	ILE168	5.49944	
BAY-876	HIS160	2.24953	Hydrogen Bond; Halogen
	SER324	2.50003	
	ALA392	2.56671	Hydrogen Bond
	GLU380	2.63907	
	HIS160	2.51892	
	SER324	2.15405	
	PRO385	2.50789	
	TRP388	2.69076	
	THR321	3.3195	Halogen
	SER324	3.69817	
	GLN161	2.80541	Hydrophobic
	PRO385	2.17417	
